# Topological protection of optical skyrmions through complex media

**DOI:** 10.1038/s41377-024-01659-z

**Published:** 2024-11-22

**Authors:** An Aloysius Wang, Zimo Zhao, Yifei Ma, Yuxi Cai, Runchen Zhang, Xiaoyi Shang, Yunqi Zhang, Ji Qin, Zhi-Kai Pong, Tádé Marozsák, Binguo Chen, Honghui He, Lin Luo, Martin J. Booth, Steve J. Elston, Stephen M. Morris, Chao He

**Affiliations:** 1 Department of Engineering Science, University of Oxford, Oxford, UK; 2 Guangdong Research Center of Polarization Imaging and Measurement Engineering Technology, Institute of Biopharmaceutical and Health Engineering, Tsinghua Shenzhen International Graduate School, Tsinghua University, Shenzhen, China; 3 College of Engineering, Peking University, Beijing, China

**Keywords:** Solitons, Optical physics

## Abstract

Optical Skyrmions have many important properties that make them ideal units for high-density data applications, including the ability to carry digital information through a discrete topological number and the independence of spatially varying polarization to other dimensions. More importantly, the topological nature of the optical Skyrmion heuristically suggests a strong degree of robustness to perturbations, which is crucial for reliably carrying information in noisy environments. However, the study of the topological robustness of optical Skyrmions is still in its infancy. Here, we quantify this robustness precisely by proving that the topological nature of the Skyrmion arises from its structure on the boundary and, by duality, is resilient to spatially varying perturbations provided they respect the relevant boundary conditions of the unperturbed Skyrmion. We then present experimental evidence validating this robustness in the context of paraxial Skyrmion beams against complex polarization aberrations. Our work provides a framework for handling various perturbations of Skyrmion fields and offers guarantees of robustness in a general sense. This, in turn, has implications for applications of the Skyrmion where their topological nature is exploited explicitly, and, in particular, provides an underpinning for the use of optical Skyrmions in communications and computing.

## Introduction

Since the discovery of the magnetic Skyrmion, there has been a growing interest in the use of topologically non-trivial states in the storage of information^1–3^. In particular, the discretized and particle-like nature of magnetic Skyrmions makes them robust to perturbations^4–6^ and hence a promising option for future memory^7^ and logic devices^8,9^. In recent years, Skyrmions have also been predicted and observed in spatially varying polarization fields^10–24^, and this has opened the doors to the potential use of such topologically non-trivial states in the transport of information^25^. In particular, optical Skyrmions have many important properties suited for high-density data applications^15,25^, namely (1) the ability to interface with digital information given their integer-valued topological number, (2) the capacity for multiplexing given the independence of spatially varying polarization to other dimensions such as amplitude, phase (including structured phase) and wavelength, and (3) the possibility to store arbitrary large integers within a single localized field.

Most importantly, there is a compelling heuristic coming from the topological stability of the magnetic Skyrmion that strongly suggests we should expect similar robustness to exist in the optical domain. The argument goes as follows. Given a spatially varying field ${\mathcal{S}}(x,y)$ taking values in the 2-sphere *S*^2^, suppose one can guarantee that the following integral equation^26^ evaluates to an integer

1 $$N=\frac{1}{4\pi }\iint \,{\mathcal{S}}\cdot \left(\frac{\partial {\mathcal{S}}}{\partial x}\times \frac{\partial {\mathcal{S}}}{\partial y}\right)dxdy$$

then, since the integrand in the equation above varies smoothly with respect to ${\mathcal{S}}$, the mapping ${\mathcal{S}}\mapsto N$ must also, in somewhat imprecise language, vary smoothly with ${\mathcal{S}}$. However, as *N* takes values in the integers, we are left with the remarkable property that smoothly deforming ${\mathcal{S}}$ cannot change the topological number of the system, for it is impossible to smoothly transition from one integer to another without also passing through every number in between. This then presents an important question on the topological robustness of optical Skyrmions: under what conditions can we guarantee that the above integral equation evaluates to a meaningful integer for a given spatially varying polarization field?

Putting aside integrality for the moment, the topological robustness established by the argument above is the primary motivation for investigating the use of optical Skyrmions as a method of reliably transmitting data through noisy channels, such as in communications and computing. The key property of electromagnetic fields at play here is the ellipticity of the underlying Helmholtz equation (in the sense of PDE theory), which guarantees the smoothness of the electric field in propagation^27^. This smooth deformation of the electric field along its direction of propagation then descends onto a smooth deformation of polarization fields, which excluding technical subtleties we mention later, establishes a constant topological number in propagation even as the transverse polarization profile changes. Moreover, this conclusion remains largely unchanged for optical fields propagating in media with continuously varying material parameters, which suggests a degree of robustness against aberrations.

There are, however, several challenges in turning the above into a formal argument, the most problematic being that the Skyrmion number integral is not always guaranteed to be integer-valued in the optical setting^11,23,24^. To elaborate, magnetic Skyrmions are naturally topological due to a preferred alignment of dipoles in the far field, which we later show to be precisely the correct mathematical condition for the Skyrmion number integral to evaluate to an integer. On the other hand, electromagnetic fields do not have a naturally preferred polarization state at infinity unless specifically designed in that way. Moreover, polarization singularities arising from zeros of the electromagnetic field are also volatile and difficult to control. These factors contribute to the existence of “fractional” phenomena in optical fields that otherwise cannot be observed in magnetic spin textures, where the above integral equation evaluates to a non-integer. Such fractional phenomena have important implications for the robustness of the optical Skyrmion, wherein the usual argument for topological stability breaks down^23^. To date, investigations into the topological robustness of spatially varying polarization fields with integer (and non-integer) Skyrmion numbers are still in their infancy, and there is scope to develop a more complete theory that elucidates the conditions that guarantee topological protection.

Motivated by the above, we give a precise and purely topological definition of the Skyrmion that clarifies the properties of an *S*^2^-valued field that allow for an integer topological number to be defined, and from this, show that the topological character of the Skyrmion is derived from its boundary. We then present a completely general framework for handling perturbations that applies to all *S*^2^-valued fields, including optical, magnetic Skyrmions, etc., which builds on recent pioneering topological robustness results established in non-local quantum Skyrmions^28,29^, and the propagation of optical Skyrmions through homogeneous retarders^30,31^. These abstract results are particularly significant as they can be applied to Skyrmions across all domains, which, in addition to optics and photonics, include condensed matter physics^1,4,32,33^, cosmology^34^, fluid dynamics^35^, soft matter physics^36^, acoustics^37,38^, and more^28,39,40^.

We then specialize our theory to paraxial Skyrmions from which a topological duality between light and matter emerges, namely that the boundary conditions satisfied by the Skyrmion are also the required boundary conditions of material parameters that guarantee topological protection. Lastly, we present experimental results that validate the robustness of the Skyrmion number as the field propagates through a complex optical system containing both typical polarization aberrations from common optical elements and perturbations mimicking more extreme situations in the real world. Our work gives new insight into the topological robustness of the optical Skyrmion and paves the way for their use in high-density data applications such as optical communications and photonic computing.

## Results

### Role of the boundary in skyrmions

As mentioned in the introduction, the lynchpin to establishing topological robustness is exhibiting conditions that guarantee the integer-valued nature of the Skyrmion number integral. We demonstrate in Materials and Methods 1 and 2 that the boundary conditions of the field determine when this is true, and we refer to fields that can be given topological character in this way *compactifiable*, similar in spirit to the Uhlenbeck compactification^41^ process in the construction of BPST instantons^42^.

**Fig. 1:**
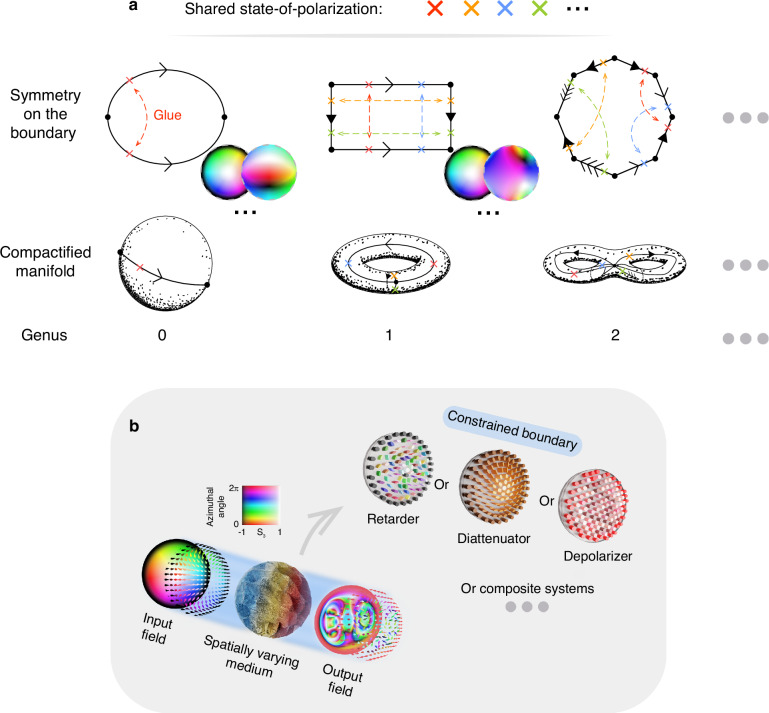
**Definition of Skyrmions and their interaction with various polarization aberrations**. **a** The various symmetry properties of the field on its boundary that allow for compactification and the corresponding compactified manifold. The symmetry properties are shown here via polygons, with arrows indicating pairwise identification of different edges oriented in the direction of the arrow. Notice that certain symmetries allow the field to be tesselated, which gives topological character to periodic structured fields. As mentioned in the main text, due to the topological nature of compactification, one can freely deform this polygon and, in the case of unbounded fields, one can consider the boundary at infinity. Possible Stokes fields satisfying the relevant symmetries are also shown. It is worth noting that a field with a constant value on its boundary satisfies every symmetry property simultaneously, however, there exist fields, non-uniform on their boundary, that can also be compactified, as shown in the figure. Throughout this paper, Stokes fields are depicted using hue to signify azimuthal angle $\tan \theta ={s}_{2}/{s}_{1}$ and saturation to represent height *s*_3_ (similar to ref. ^15^). **b** Schematic depicting the effects of a complex spatially varying medium on an incident optical Skyrmion field. In this paper, systems exhibiting spatially varying retardance, diattenuation, depolarization, and combinations of the aforementioned are carefully considered. Note that the boundary of the incident field and the corresponding aberrations are of key importance in guaranteeing topological protection. Throughout this work, fields with a constant value on their boundaries are considered for physical reasons expanded on in the main text

In simple terms, a field is compactifiable if its domain can be viewed as a compact and orientable surface, which via the classification theorem for compact surfaces, is characterized entirely by its genus, or more intuitively, the number of “holes” of the surface^43^. From standard polygonal constructions of topological surfaces, one way to understand compactifiability is as a symmetry property of the boundary, where the appropriate symmetries allow one to “glue” the boundary together into a surface. Figure 1a gives examples of different symmetries that allow for compactification.

Notice that while a field constant on its boundary satisfies all possible symmetries, there also exist fields, non-uniform on their boundary, for which compactification holds (See Fig. 1a). Moreover, while polygons are used to depict these symmetries, the topological nature of compactification allows deformations of these polygons. In the case of optical fields, which need not necessarily be confined, we can extend our considerations to symmetries of the boundary at infinity.

Notably, if a field ${\mathcal{S}}:U\longrightarrow {S}^{2}$ is compactifiable, then it necessarily satisfies an uncountable number of non-trivial integral equations

2 $${\int_{U}}{{\mathcal{S}}}^{* }\omega =\,{\text{deg}}\,{\mathcal{S}}{\int_{{S}^{2}}}\omega$$

one for every *ω* ∈ *Λ*^2^(*S*^2^). The integer-valued nature of the usual Skyrmion number integral equation is then a consequence of the above. We also show in Materials and Methods 3 that compactifiable fields satisfy a natural notion of homotopy invariance from which the Skyrmion derives its topological robustness. Lastly, note that while the usual integral equation evaluates to an integer for a Skyrmion, the converse need not be true, and just knowing that equation (1) evaluates to an integer for a specified field gives no clear topological information about the underlying field and no clear indication of whether the integral equation is invariant under homotopies of the field.

With the notion of compactifiability, we can revisit the arguments for the topological robustness of optical Skyrmions presented in the introduction. As before, the ellipticity of the Helmholtz equation provides the necessary regularity for the natural propagation of electric fields to descend onto homotopies of polarization fields. If, further, the polarization field has the necessary boundary conditions to be compactifiable on every transverse plane, then the integer-valued nature of the Skyrmion number integral along these planes combined with the usual smoothness argument once again establishes topological protection in propagation.

Notice that our theory is a generalization of the usual methods applied to paraxial Skyrmion beams^11,24^ that rely on a superposition of Laguerre-Gaussian (LG) modes and proves topological protection for any complex superposition of modes provided that there are no zeros of the field and that compactification holds (for example, if the polarization state approaches a constant value at infinity). Moreover, this approach relies on neither a specific modal decomposition, whose linear nature is intrinsically incompatible with the decidedly non-linear nature of topology, nor theoretical computations of Skyrmion numbers along the transverse plane, which quickly become intractable as the number of superposed modes increases.

We give a more formal description of this procedure for establishing topological robustness in Materials and Methods 3. Note also that our approach does not reference any particular property of optical fields and can, therefore, be used to establish the preservation of the Skyrmion number in any number of settings beyond paraxial optical Skyrmions, including optical Skyrmions in evanescent fields, magnetic Skyrmions, etc. Moreover, the procedure we have introduced is, in some sense, both necessary and sufficient in proving topological protection. This is because if we compactify, as we usually do, onto the 2-sphere, then the Hopf degree theorem provides the converse statement that if topological protection holds, then there exists some way of demonstrating this fact using the procedure in Materials and Methods 3.

### Specializing to optical aberrations and the paraxial optical skyrmion

To further demonstrate the utility of our approach, we specialize our methodology to paraxial optical Skyrmions and show that in this case, the Skyrmion number remains conserved across a broad range of important optical aberrations including spatially varying retarders, diattenuators, specific classes of depolarizing systems, and more generally, any complex system composed of such elements (see Fig. 1b for a pictorial representation of the systems under consideration and Materials and Methods 4–7 for detailed derivations). Note that we exclude strongly depolarizing and absorbing media in our considerations, which can disrupt topological character by introducing polarization singularities into the field. However, since such aberrations are not prevalent in optical systems, our theory remains applicable to the vast majority of cases, with extensions to more severe aberrations an exciting avenue to explore.

In each case, we show that the compactifiability of material parameters is one of the conditions needed for ensuring topological protection, with a general tolerance to perturbations away from the boundary provided they remain smooth. Our result is a consequence of the compactification formalism introduced, which makes explicit the fact that the topological nature of the Skyrmion is derived from its boundary. This is particularly important for paraxial optical Skyrmions, whose natural boundary is at infinity due to the infinite extent of the fundamental solution to the Helmholtz equation and, therefore, remains unaffected by the necessarily finite aberrations along the direction of propagation.

We would also like to emphasize that retarders, diattenuators, and depolarizers are abstract mathematical concepts^44^ that can be used to describe polarization aberrations and are formalisms that can be applied to a large number of optical components^44,45^. Since such polarization aberrations alter the field of a paraxial Skyrmion beam, potentially affecting the topological information carried, it is important to study and quantify the topological robustness of the Skyrmion against such aberrations.

More concretely, important aberrations that can be described in this way include the Fresnel effect from reflections and refractions, anisotropic absorptions of coatings, birefringence, depolarization induced by imperfections of optical components, etc. The typical levels of such aberrations have been studied before and can be characterized by polarimetry or ellipsometry^46–50^. In the majority of cases, they are spatially varying smooth aberrations with weak values on their boundary. This observation links the abstract mathematical notion of compactifiability to physical aberrations in real optical systems and provides a heuristic justification for the choice of aberrating elements and generated Skyrmion beams in the experiments that we describe below. In these experiments, we validate the robustness of the optical Skyrmion against various optical aberrations, including both aberrations from common optical elements as well as atypical aberrations that mimic more extreme perturbations that exist in real-world settings. Lastly, we reiterate that the aberrations considered in this section have been selected due to their physical significance, and are merely specific examples of perturbations that can be handled by the generality of our theory.

### Experiments

To back up our theoretical work, we present experimental results that validate the robustness of the Skyrmion number as the field propagates through a complex optical system. In our experiments, we synthesized Skyrmions with degrees ranging from 1 to 10 and propagated these Skyrmions through a complex optical system with multiple aberrating elements. We then measured the Stokes field using standard Stokes polarimetry and computed the Skyrmion number integral from our measurements using a technique described below. The target Stokes fields generated in our experiments take the form

3 $$\begin{array}{lll}\left(\begin{array}{l}{S}_{1}\\ {S}_{2}\\ {S}_{3}\end{array}\right)&=&\left(\begin{array}{l}\sqrt{1-f{(r)}^{2}}\cos n\theta \\ f(r)\\ \sqrt{1-f{(r)}^{2}}\sin n\theta \end{array}\right)\\ f(r)&=&\left\{\begin{array}{l}\exp \left(1-\frac{1}{1-{r}^{2}}\right)-1,\quad{0}\le \,{r}\le \,{1}\\ \qquad\qquad\qquad\,\,\,\,{-1},\quad{1}\, < \,{r}\end{array}\right.\end{array}$$

where *n* is the target degree of the generated Skyrmion and *r* is a normalized radius. This field is realized using a cascade of two liquid crystal spatial light modulators (LC-SLM) aligned at a 45^∘^ angle to each other as shown in Fig. S5, and where the same number of pixels is used in generation for all experiments. For a detailed description of the pixelated arbitrary state-of-polarization generation technique adopted, see refs. ^30,51^. Note also that this particular choice of Stokes field was made as it is everywhere differentiable, even as we transition from the spatially varying part when *r* ≤ 1 to the homogeneous one when *r* > 1. As a consequence, we may interpret the generated field as a Skyrmion, in the sense of compactifiability, for any open set *U* containing the unit ball centered at the origin. This choice of boundary condition also matches the material properties of the various aberrations used, which as explained in Materials and Methods 4–7, is the duality condition in guaranteeing topological protection.

**Fig. 2:**
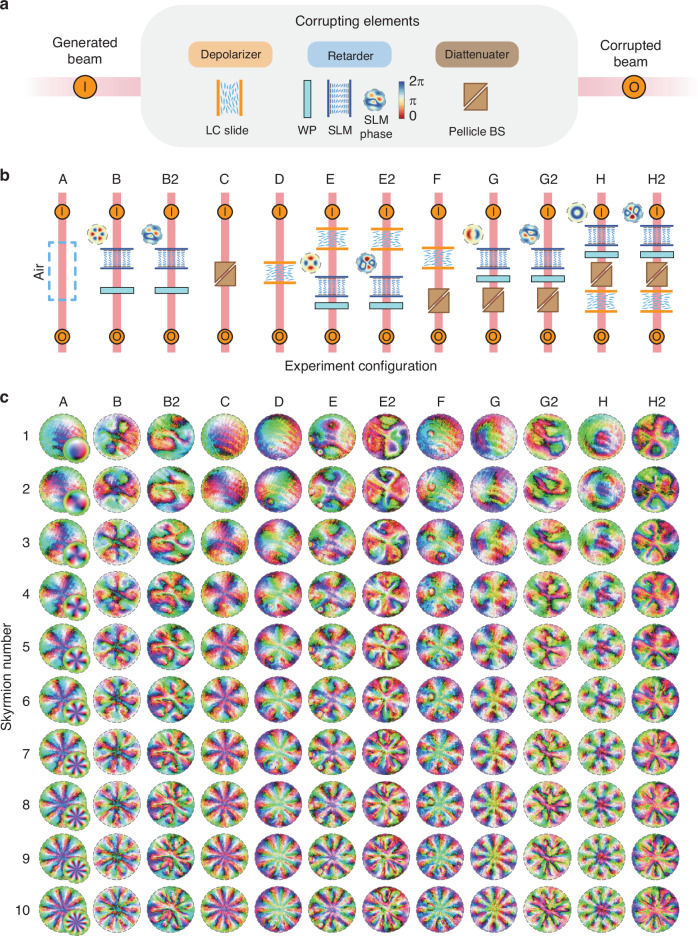
**Experiment assembly and measured Stokes fields**. **a** The various corrupting elements used in testing, and the class of media they belong to. In experimentation, the corrupting components are placed immediately after the beam generator. Here, I indicates the input beam and O the output beam. **b** The order of components and SLM phase patterns used in testing. **c** The measured Stokes fields for different experiment configurations and input Skyrmion numbers. Simulated Stokes fields for configuration A are also shown

**Fig. 3:**
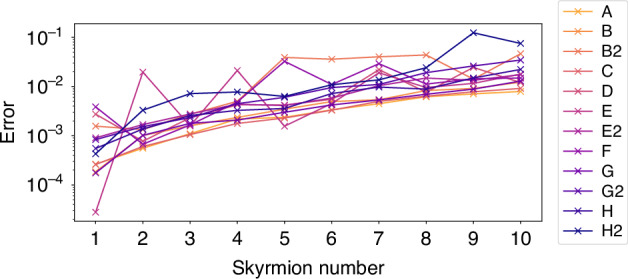
**Computed Skyrmion number and associated error**. The associated error as defined in equation (4) across all configurations

The corrupting elements in our experiments consist of a cascade of up to four components, including an LC-SLM and variable waveplate that together act as a spatially varying retarder, a pellicle beamsplitter that possesses a high diattenuation of approximately 0.25, and a nematic LC mixture sandwiched between two glass substrates that simultaneously acts as a retarder while also weakly depolarizing incident light (Fig. 2a). Importantly, these elements mimic the effects of common aberrations found in optical systems as well as stronger, more atypical aberrations that may exist in the real world. The different aberrating elements and their relative placements in testing (from A to H2) are shown in Fig. 2b, the observed Stokes fields measured in experiments shown in Fig. 2c, and the error in computed Skyrmion numbers shown in Fig. 3. We also provide a quantitative assessment of the level of aberrations present in select configurations via Mueller matrices in Fig. S6.

We would like to highlight three important technicalities. Firstly, in the synthesis of our fields, a relatively small fraction of the SLM is dedicated to the spatially varying portion of the field while the majority of pixels are used to impose boundary conditions. Due to the homotopy invariance of the degree, the demands on the resolution required to synthesize the spatially varying part of the field are fairly gentle. Indeed, the homotopy class of degree *n* functions is an enormous collection of different maps, and heuristically, as long as the produced field qualitatively resembles the target field, they are likely to belong to the same homotopy class. Notice from Fig. 2c that although the fidelity of our synthesized field is limited by imperfections, especially of the SLM, the resulting Skyrmion number remains close to the target Skyrmion number. As an aside, the technique we have adopted here for Skyrmion generation is capable of producing Skyrmions of significantly higher order than the usual superposition of LG modes.

Secondly, in all experiments, linear interpolation followed by a Gaussian filter and projection onto *S*^2^ is applied in post-processing before the numerical evaluation of the Skyrmion number. This achieves two important goals. Firstly, smoothing helps limit the effects of noise on computations of partial derivatives, and secondly, smoothing homogenizes perturbations that have occurred in the boundary, therefore allowing for compactification. The latter is true because, by design, both our field and aberrations have been chosen to be relatively uniform for *r* > 1. Note also that our post-processing method is permissible from the perspective of topology as Gaussian filtering—provided the standard deviation of the kernel is not too large as to cause points to cross hemispheres—can be regarded as a homotopy of the field by linearly interpolating standard deviation from 0.

Lastly, Fig. 3 shows the error defined by

4 $${\text{error}}={\text{Computed}}\, {\text{Skyrmion}}\, {\text{number}}-{\text{Target}}\, {\text{Skyrmion}}\, {\text{number}}$$

across different target Skyrmion numbers and experiment configurations. While the computed error is reasonably small, notice that there is a general trend for error to increase as Skyrmion number increases. One possible explanation is that numerical errors in differentiation and integration typically scale with the sup norm of suitable partial derivatives. Therefore, increasing the complexity of the field necessarily impacts the accuracy that can be achieved in numerical integration. Another possibility is that due to the limitations of the SLMs, such as discontinuities along pixels and limited resolution, the topological properties of Skyrmions of high order are less stable and, therefore, more affected by aberrations.

Despite possible difficulties of resolving Skyrmions with a large Skyrmion number, our experiments show that for degrees up to 10, the computed Skyrmion number from polarimetric measurements—even in the presence of aberrating elements—remains close to the target Skyrmion number. With a maximum error of about 0.1, our results demonstrate the robustness of the topology of optical Skyrmions and support the use of Skyrmions in high-density data applications such as optical communications and photonic computing.

Lastly, there is interesting scope for investigating the limit for which topological protection fails to hold, but due to the already lengthy nature of this work, we defer such an investigation to a future paper, noting that a rigorous treatment of the subject will require proving both necessary and sufficient topological conditions for equation (2) to hold.

## Discussion

First and foremost, we would like to emphasize that there exist complex spatially varying media for which topological protection does not hold. A direct example comes from specially designed gradient index systems, where one may explicitly show this to be true (see Supplementary Note 8). The reason this system does not offer topological protection is that its material parameters are not compactifiable. As a result, an incident Skyrmion loses its compactifiable structure as it propagates through the medium, and even if the resultant field is a Skyrmion, propagation through the system does not descend onto to homotopy in the sense of Materials and Methods 3.

In this paper, we do not proclaim that the optical Skyrmion is resilient to all perturbations; rather, we have tried to develop a definition of the Skyrmion and a corresponding framework for establishing results of topological robustness that can be derived from this definition. There is, however, significant scope to expand on these results. Whether or not, and to what extent, topological protection holds remains an important question in the study of optical Skyrmions, and both positive and negative results help elucidate fundamental properties of the optical Skyrmion. Moreover, negative results may even be helpful in the context of computing, where manipulation of the Skyrmion number can be used to perform integer arithmetic.

We would also like to stress that the ability to generate Skyrmions of such high order is peculiar to the optical setting. Unlike magnetic Skyrmions, which are topological solitons in a true sense and have a notion of energy stability, such a concept has no analog in the optical domain. This energy stability in magnetic Skyrmions gives rise to a physical interpretation of topological protection beyond the abstract mathematical one stated in this paper, namely energy input is necessary to transition from one stable topological state to another. However, the only interpretation accessible to the optical Skyrmion is the mathematical one. While this lack of an energy barrier has implications for the topological stability of optical Skyrmions, it also has significant advantages in terms of data density, where energy constraints do not impede the formation of high-order Skyrmions as we demonstrate in our experiments. In this sense, the theory of optical Skyrmions is not so much the study of a naturally topological system but rather about the ability to embed topological character into electromagnetic fields through techniques of structured light and about investigating the limits of this topological character.

Lastly, we consider here the significance of the assumptions of smoothness and compactifiability we have made in establishing our theory. Note firstly that smoothness is a condition we introduced to ease our discussion of the topic and can, in fact, be greatly weakened. Indeed, most of what we have done holds directly for *C*^1^ functions, but we can even generalize to continuous functions by swapping out the De Rham cohomology for singular cohomology. In this case, the integral equation for the Skyrmion number may not be well-defined as partial derivatives need not necessarily exist, but general density results guarantee that a continuous ${\mathcal{S}}$ is, in a suitable sense, close to some smooth ${{\mathcal{S}}}^{{\prime} }$ of equivalent degree for which the integral equation holds. Importantly, as continuous functions are a much larger class than smooth functions, being able to handle continuous maps grants greater applicability of our theory to the real world.

Compactifiability, on the other hand, is nonoptional, and while many vector beams naturally satisfy this condition, it is still of practical and theoretical interest to extend the notion of the Skyrmion to a broader class of functions. Here, we propose one such way.

The most obvious way of generalizing the Skyrmion is to simply drop the compactifiable condition and declare

5 $$\begin{array}{lll}\displaystyle{\text{the}}\, {\text{Skyrmion}}\, {\text{Number}}\, {\text{of}}\,\,{\mathcal{S}}\equiv \,\text{SkyN}\,({\mathcal{S}})& = & \displaystyle {\int}_{U}{{\mathcal{S}}}^{* }{\omega }_{0}\\&=& \displaystyle\frac{1}{4\pi }{\iint }_{U}S\cdot \left(\frac{\partial S}{\partial x}\times \frac{\partial S}{\partial y}\right)dxdy\end{array}$$

In doing so, we gain the ability to assign arbitrary fields with a Skyrmion number but lose two important properties. Firstly, $\,\text{SkyN}\,({\mathcal{S}})$ is no longer guaranteed to be an integer, and secondly, $\,\text{SkyN}\,({\mathcal{S}})$ can no longer be interpreted as the degree of a smooth map. Therefore, the Skyrmion number, as defined above, is not a topological number in the true sense, and the cost of this generality is that we muddy the topological interpretation of the Skyrmion. Nonetheless, we can still proceed as in Supplementary Note 1.

We call Skyrmions defined in this more general way “fractional Skyrmions” and discuss the manners in which their topological nature can be interpreted in Supplementary Note 2. Note that while other works have also brought up notions of a fractional Skyrmion^23^, the interpretations we give in Supplementary Note 2 prescribe these fields a precise topological character and, importantly, a notion of homotopy invariance that can be used to establish conditions, albeit weaker, for the Skyrmion number integral to remain unchanged. In the case of optical fields, we can, as before, give sufficient conditions for the preservation of the Skyrmion number through spatially varying retarders, diattenuators, depolarizing media, and their combinations. In Supplementary Note 3, we flesh out the mathematical details of this argument. Lastly, notice that the abstract results we prove here are directly applicable to important real-world situations where the boundary conditions of the field are sufficiently constrained, such as propagation in a conducting waveguide.

To conclude, in this paper, we proposed a topological definition of the Skyrmion that clarifies conditions for the Skyrmion number integral to evaluate to an integer, namely compactifiability, and made clear the properties of the underlying physics that allows such topological structures to propagate in electromagnetic fields, namely the ellipticity of the Helmholtz equation. Using our new definition, we establish general procedures for handling perturbations of Skyrmion fields, and demonstrated, both theoretically and experimentally, the robustness of an optical Skyrmion’s topological structure against important polarization aberrations.

As a concluding remark, we emphasize once again that the formalism of compactification implies the existence of *S*^2^-valued fields, not uniform on the boundary, for which an integer topological number can be defined. While the prevailing approach to optical Skyrmions has been to consider fields constant on their boundaries, there is certainly room to explore topological textures with more complicated symmetries.

We believe the work presented in this paper establishes a solid foundation for further investigations into the topological protection of Skyrmions and hope our findings can spur further developments in the understanding of Skyrmions across all domains, including optics and photonics, condensed matter, cosmology, fluid dynamics, acoustics, soft materials, and more.

## Materials and methods

### Defining the skyrmion

The central tool in understanding the topology of the Skyrmion is the De Rham cohomology^52^, which is the mathematical theory that allows one to prescribe spatially varying fields taking values in *S*^2^, such as polarization fields, topological character via an integral equation. It is a well-known result in multivariable calculus that on a contractible open subset of ${{\mathbb{R}}}^{n}$, if a *k*-form *ω* satisfies *dω* = 0, then there necessarily exists a (*k* − 1)-form *η* so that *dη* = *ω*. This can be understood as a generalization of the more commonly seen facts in physics that a divergence-free vector field can be written as the curl of a vector potential, and a curl-free vector field can be written as the gradient of a scalar potential. Therefore, if there exists a *k*-form *ω* satisfying *dω* = 0 but no corresponding (*k* − 1)-form *η* so that *dη* = *ω*, this gives us topological information about the underlying space, namely that it cannot be contractible. The De Rham cohomology expands this idea by introducing a collection of algebraic objects that can be assigned to any smooth manifold *X* called its De Rham cohomology groups ${H}_{{\rm{de}}\,{\rm{R}}}^{k}(X)$ and whose algebraic structure subtly captures information about how the underlying space fails to be contractible.

Given a compact, connected, oriented, smooth, *n*-dimensional manifold *X*, one can show using Stokes’ theorem that the top cohomology group is isomorphic to ${\mathbb{R}}$ as a vector space via the map $\omega \mapsto \int_{X} \omega$. Given two such manifolds *X* and *Y*, and a smooth map $f:X \longrightarrow Y$ between them, the naturally induced map in cohomology given by pullback is then a ${\mathbb{R}}$-linear map ${f}^{\#}:{H}_{{\rm{de}}\,{\rm{R}}}^{n}(Y)\cong {\mathbb{R}}\longrightarrow {H}_{{\rm{de}}\,{\rm{R}}}^{n}(X)\cong {\mathbb{R}}$. As the only ${\mathbb{R}}$-linear map from ${\mathbb{R}}$ to itself is of the form $x \mapsto cx$ for some $c\in {\mathbb{R}}$, we may assign to every such *f* a real number ${\text{deg}}\,{f} := c$. Using an argument invoking the regular value theorem, one can further show that the degree of any map takes values in the integers. The homotopy invariance of the degree is then the key mathematical concept that captures our intuitions of what topological robustness to perturbations should be.

With the above machinery on hand, we are in the position to define the Skyrmion. Given a field that takes values in the 2-sphere, checking against the hypotheses in the definition of the degree, we see that the target space of the field is already a compact, connected, oriented, smooth, 2-dimensional manifold. The domain of the field is, however, not naturally of this form. Therefore, extra work is required to “non-trivialize” the domain of the field. If we are interested in fields that occupy three dimensions, there is an immediate solution by restricting to a submanifold of ${{\mathbb{R}}}^{3}$ with the required properties. Defined like this, the Skyrmion can be regarded, topologically, as a strict higher dimensional generalization of orbital angular momentum, which derives its topological character in exactly this way, except by restricting phase to a suitable one-dimensional compact and connected submanifold of ${{\mathbb{R}}}^{2}$.

However, the drawback of using the above approach is that the Skyrmion becomes a fundamentally three-dimensional object rather than having the more commonly understood planar structure. In the context of optical fields, such an interpretation of the Skyrmion also loses the natural notion of propagation, and we would much rather define the Skyrmion by considering polarization fields when restricted to the transverse plane. Here, the mathematics inevitably becomes more technical. Instead of simply restricting to a submanifold with the necessary properties, one must extend the trivial two-dimensional plane into a non-trivial manifold. However, this process, which we formalize in Materials and Methods 2, restricts the fields that can and cannot be considered a Skyrmion. We call fields compatible with the extension of its underlying domain compactifiable and note that the boundary conditions of the field determine whether compactification holds. For instance, a field that approaches a constant value on its boundary can be compactified through stereographic projection, while a periodic field can be compactified by projection onto the torus ${{\mathbb{R}}}^{2}/{{\mathbb{Z}}}^{2}\cong {{\mathbb{T}}}^{2}$.

The significance of what are ostensibly just mathematical technicalities is that they clarify precisely what properties of the field allow for the definition of an integer-valued topological number, which, as mentioned in the introduction, is the lynchpin in establishing topological robustness. Moreover, from our definition, we can show that any compactifiable ${\mathcal{S}}:U\longrightarrow {S}^{2}$ satisfies an uncountable collection of non-trivial integral equations given by

6 $${\int_{U}}{{\mathcal{S}}}^{* }\omega =\,{\text{deg}}\,{\mathcal{S}}{\int_{{S}^{2}}}\omega$$

for every *ω* ∈ *Λ*^2^(*S*^2^), of which the usual integral equation is just a special case (see Supplementary Note 4). In fact, satisfying the usual integral equation does not capture any topological structure of the underlying field, and it is easy to construct a field where the usual integral equation evaluates to an integer but not an uncountable number of other integral equations that we expect a truly topological field to satisfy (take, for example, a meron of second-order vorticity). In this sense, defining the Skyrmion through the integral equation is simply nonsensical from the perspective of topology.

### Compactifiability

Suppose we have an field ${\mathcal{S}}:U\longrightarrow {S}^{2}$ defined on an open subset *U* of ${{\mathbb{R}}}^{2}$. If there exists a compact, connected, oriented 2-dimensional manifold *X* and a smooth map *ψ*: $U \longrightarrow X$ satisfying the following properties:

1. *ψ* is an orientation preserving diffeomorphism of *U* onto its image,
2. *ψ*(*U*) is a dense subset of *X* with full measure,
3. ${\mathcal{S}}\circ {\psi }^{-1}$ extends via continuity to a smooth map $\tilde{{\mathcal{S}}}$ on all of *X*,

we call ${\mathcal{S}}$ compactifiable, $\tilde{{\mathcal{S}}}$ its compactification, and prescribe the field ${\mathcal{S}}$ with a Skyrmion number $\,{\text{deg}}\,{\mathcal{S}}:=\,{\text{deg}}\,\tilde{{\mathcal{S}}}$, where we understand $\,{\text{deg}}\,{\tilde{{\mathcal{S}}}}\in {\mathbb{Z}}$ as the degree of a smooth map originating from De Rham cohomology. The topological nature of the Skyrmion then results from the fact that two Skyrmion fields ${\tilde{{\mathcal{S}}}}$ and ${\tilde{{\mathcal{S}}}}\!{\scriptstyle{\prime}\atop}$ have the same degree if they are smoothly homotopic. Note that for fields with compact domains, a more natural way of compactification is via quotients. However, such an approach is not applicable to ${{\mathbb{R}}}^{2}$, which is the most natural domain to consider in many cases. In fact, there is no loss of generality in adopting the notion of compactification presented here as a field defined on a compact set that descends onto a surface through a suitable quotient can also be compactified, in the sense we have defined above, by considering the restriction of the field onto the interior of its domain.

To derive the usual Skyrmion number integral from this definition, let $\iota :{{S}^{2}}\hookrightarrow {{\mathbb{R}}}^{3}$ be the inclusion, *ω*_0_ ∈ *Λ*^2^(*S*^2^) the normalized volume form ${\omega }_{0}={\frac{1}{4\pi }\iota^{\ast}}({x}^{1}d{x}^{2}\wedge d{x}^{3}-{x}^{2}d{x}^{2}\wedge d{x}^{3}+{x}^{3}d{x}^{1}\wedge d{x}^{2})$ where *x*^1^, *x*^2^ and *x*^3^ are the standard coordinate functions on ${{\mathbb{R}}}^{3}$, and $S:=\iota \,{\circ}\, {\mathcal{S}}$. If *x* and *y* are the standard coordinate functions on *U*, then

7 $$\begin{array}{l}\,{\text{deg}}\,{\mathcal{S}}:={\int_{X}}{\tilde{{\mathcal{S}}}}\!{\scriptstyle{*}\atop}{\omega }_{0}={\int_{\psi (U)}}{\tilde{{\mathcal{S}}}}\!{\scriptstyle{*}\atop}{\omega }_{0}={\int}_{U}{\psi }^{* }({\tilde{{\mathcal{S}}}}\!{\scriptstyle{*}\atop}{\omega }_{0})\\ \qquad\qquad\qquad\qquad\qquad\qquad\quad\,\,\,={\int}_{U}{(\tilde{{\mathcal{S}}}\circ \psi )^{*}}{\omega }_{0}={\int_{U}}{{\mathcal{S}}}^{* }{\omega }_{0}=\frac{1}{4\pi }{\iint_{U}}S\cdot \left(\frac{\partial S}{\partial x}\times \frac{\partial S}{\partial y}\right)dxdy\end{array}$$

which is the Skyrmion number integral in its usual guise (see Supplementary Note 4). Note that the technical conditions (1) and (2) are precisely the conditions needed to pullback *ω* from *S*^2^ to *U*, with the density of *ψ*(*U*) giving the first equality above and *ψ* being an orientation preserving diffeomorphism giving the second. Notice also that although we required the existence of *ψ* for compactification, the Skyrmion number itself is independent of the choice of *ψ*. The Skyrmion number is, therefore, a genuine characteristic of the field.

### A homotopy approach to topological protection

Using the notion of compactifiability, we can determine the effects of any perturbation on the topology of a Skyrmion using the following simple procedure.

1. Write the perturbed field ${{\mathcal{S}}}^{{\prime} }:U\longrightarrow {S}^{2}$ in the form ${{\mathcal{S}}}^{{\prime} }(p)=f({\mathcal{S}}(p),{\alpha }_{1}(p),\ldots ,{\alpha }_{k}(p))$ for some smooth function *f* and smoothly varying parameters *α*_*i*_, *i* = 1, …, *k*, that characterize the perturbation.
2. Construct a smooth homotopy from ${\tilde{{\mathcal{S}}}}^{{\prime} }$ to a map of the form $\bar{f}\circ \tilde{{\mathcal{S}}}$ for some smooth ${\bar{f}}$. Then, by the smooth homotopy invariance of the degree, we have $\,{\text{deg}}\,{{\mathcal{S}}}^{{\prime} }:=\,{\text{deg}}\,{\tilde{{\mathcal{S}}}}^{{\prime} }=\,{\text{deg}}\,({\bar{f}}\,{\circ}\, {\tilde{{\mathcal{S}}}})=(\,{\text{deg}}\,{\bar{f}})(\,{\text{deg}}\,\tilde{{\mathcal{S}}})=:(\,{\text{deg}}\,{\bar{f}})(\,{\text{deg}}\,{\mathcal{S}})$.

The key benefit of this approach is that we can often reduce our analysis from the possibly intractable Skyrmion number integral to the much simpler computation $\,{\text{deg}}\,\bar{f}=\mathop{\sum }\nolimits_{i = 1}^{k}\,{\text{sign}}\,(\bar{f},{p}_{i})$, where $\{{p}_{1},\ldots ,{p}_{k}\}={\bar{f}}^{-1}(q)$ for any regular value *q* of $\bar{f}$, $\,{\text{sign}}\,(\bar{f},{p}_{i})=1$ if ${\bar{f}}_{* {p}_{i}}$ is orientation preserving and $\,{\text{sign}}\,(\bar{f},{p}_{i})=-1$ if ${\bar{f}}_{* {p}_{i}}$ is orientation reversing. As a technical aside, observe that in order for ${{\mathcal{S}}}^{{\prime} }$ to be a Skyrmion field, it is sufficient for $\alpha_{i} \circ \psi^{-1}$ to extend via continuity to a smooth map ${\tilde{\alpha }}_{i}$ on all of *X*. In which case, the smoothness of *f* implies that ${{\mathcal{S}}}^{{\prime} }\,{\circ}\,{\psi }^{-1}$ also extends via continuity to the smooth map ${\tilde{{\mathcal{S}}}}^{{\prime} }:X\to {S}^{2}$, ${\tilde{{\mathcal{S}}}}^{{\prime} }(x)=f(\tilde{{\mathcal{S}}}(x),{\tilde{\alpha }}_{1}(x),\ldots ,{\tilde{\alpha }}_{k}(x))$. Lastly, as mentioned in the main text, if the codomain of *ψ* is *S*^2^, then the Hopf degree theorem guarantees that topological protection holds if and only if the above procedure holds for some homotopy.

### Spatially varying retarders

Consider a spatially varying retarder characterized by its Jones matrix $e^{i\phi (p)}J(p)$, where $J: U \longrightarrow SU(2)$ represents the relative phase shift between fast and slow axes, and $\phi :U\longrightarrow {\mathbb{R}}$ the absolute phase^53^. Through our proposed methodology, we show that topological protection is guaranteed whenever *J* is smooth and compactifiable in a compatible way with the incident field, that is $J \circ \psi^{-1}$ extends via continuity to a smooth map for the same *ψ* that compactifies ${\mathcal{S}}$. We call compactifiability in this compatible way the duality condition for guaranteeing topological protection. Adopting the language introduced in Materials and Methods 3, we first write

8 $${{\mathcal{S}}}^{{\prime} }(p)=f({\mathcal{S}}(p),J(p))={A}^{T}\,\text{Spin}\,(J(p))A{\mathcal{S}}(p)$$

where Spin: $SU(2) \longrightarrow SO(3)$ is the usual Spin map

9 $$\left(\begin{array}{cc}a+bi&c+di\\ -c+di&a-bi\end{array}\right)\mapsto \left(\begin{array}{ccc}{a}^{2}-{b}^{2}-{c}^{2}+{d}^{2}&2ab+2cd&-2ac+2bd\\ -2ab+2cd&{a}^{2}-{b}^{2}+{c}^{2}-{d}^{2}&2ad+2bc\\ 2ac+2bd&2bc-2ad&{a}^{2}+{b}^{2}-{c}^{2}-{d}^{2}\end{array}\right)$$

and

10 $$A=\left(\begin{array}{ccc}0&1&0\\ 0&0&1\\ 1&0&0\end{array}\right)$$

To construct a suitable homotopy, let $\Phi :SU(2)\longrightarrow {S}^{3}\subseteq {{\mathbb{R}}}^{4}$ be the canonical diffeomorphism given by

11 $$\Phi \left(\begin{array}{cc}a+bi&c+di\\ -c+di&a-bi\end{array}\right)=\left(\begin{array}{c}a\\ b\\ c\\ d\end{array}\right)$$

As *X* is a 2-dimensional manifold and *S*^3^ a 3-dimensional manifold, $\Phi \,{\circ}\, {\tilde{J}}:X\longrightarrow {S}^{3}$ is never surjective. There therefore exists some *q* ∈ *S*^3^ not in the image of $\Phi \,{\circ}\, {\tilde{J}}$. We now define a continuous homotopy from $\Phi\, {\circ} \,{\tilde{J}}$ to the constant map taking value −*q* as follows. Let $H: X \times [0, 1] \longrightarrow S^3$ be

12 $$H(x,t)=\frac{(1-t)\Phi (\tilde{J}(x))-tq}{\parallel (1-t)\Phi (\tilde{J}(x))-tq\parallel }$$

Since $\Phi ({\tilde{J}}(x))\ne q$, $\parallel (1-t)\Phi ({\tilde{J}}(x))-tq\parallel$ is never zero, and hence *H* is well-defined. Moreover, *H* is easily seen to be continuous, and satisfies $H(x,0)=\Phi ({\tilde{J}}(x))$ and $H(x, 1) =-q$, as required. By a further application of the Whitney Approximation Theorem (see Supplementary Note 5), we can find a smooth homotopy ${\tilde{H}}:X\times (-\epsilon ,1+\epsilon )\longrightarrow {S}^{3}$ with ${\tilde{H}}(x,0)=\Phi ({\tilde{J}}(x))$ and ${\tilde{H}}(x,1)=-q$. Using this, we may smoothly homotope ${\tilde{{\mathcal{S}}}}^{{\prime} }$ to ${A}^{T}\,{\text{Spin}}\,({\Phi}^{-1}(-q))A{\tilde{{\mathcal{S}}}}$ via $F: X \times ( - \epsilon, 1 + \epsilon) \longrightarrow S^2$,

13 $$F(x,t)={A}^{T}\,{\text{Spin}}\,({\Phi }^{-1}({\tilde{H}}(x,t)))A{\tilde{{\mathcal{S}}}}(x)$$

Then, by the smooth homotopy invariance of deg, we have

14 $$\,{\text{deg}}\,{{\mathcal{S}}}^{{\prime} }:=\,{\text{deg}}\,{\tilde{{\mathcal{S}}}}^{{\prime} }=\,{\text{deg}}({A}^{T}{\text{Spin}}\,({\Phi }^{-1}(-q))A{\tilde{{\mathcal{S}}}})$$

Notice now that ${A}^{T}\,{\text{Spin}}\,({\Phi }^{-1}(-q))A{\tilde{\mathcal{S}}}$ is just ${\bar{f}}\,{\circ}\, {\tilde{\mathcal{S}}}$ where ${\bar{f}}:{S}^{2}\longrightarrow {S}^{2}$ is a rotation. Since every rotation is an orientation preserving diffeomorphism, $\,{\text{deg}}\,{\bar{f}}=1$. We therefore have

15 $$\begin{array}{lll}{\text{deg}}({A}^{T}{\text{Spin}}\,({\Phi }^{-1}(-q))A{\tilde{\mathcal{S}}})&=& {\text{deg}}\,({\bar{f}}\,{\circ}\, {\tilde{\mathcal{S}}})\\&=& (\,{\text{deg}}\,{\bar{f}})(\,{\text{deg}}\,{\tilde{\mathcal{S}}})\,=\,{\text{deg}}\,{\tilde{\mathcal{S}}}=:\,{\text{deg}}\,{\mathcal{S}}\end{array}$$

as required.

To support our mathematical proof, we numerically computed the Skyrmion number of an incident Skyrmion field after it passes through a complex spatially varying retarder. For convenience, we consider a special class of retarders given by the parametrization

16 $$J=\left(\begin{array}{cc}{\cos }^{2}(\alpha ){e}^{i\Delta /2}+{\sin }^{2}(\alpha ){e}^{-i\Delta /2}&2i\cos (\alpha )\sin (\alpha )\sin (\Delta /2){e}^{-i\delta }\\ 2i\cos (\alpha )\sin (\alpha )\sin (\Delta /2){e}^{i\delta }&{\sin }^{2}(\alpha ){e}^{i\Delta /2}+{\cos }^{2}(\alpha ){e}^{-i\Delta /2}\end{array}\right)$$

where $\alpha ,\delta :U\longrightarrow {\mathbb{R}}$ are smooth maps defining a smoothly varying fast axis $(\cos (\alpha ){e}^{-i\delta /2},\sin (\alpha ){e}^{i\delta /2})$, and $\Delta :U\longrightarrow {\mathbb{R}}$ the corresponding smoothly varying retardance. Note that spatial dependence has been suppressed here for clarity.

**Fig. 4:**
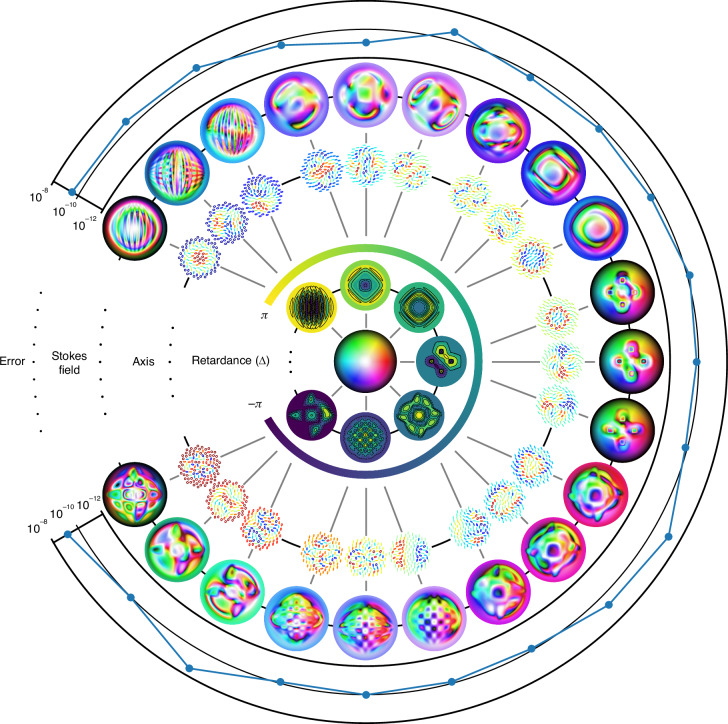
**Skyrmions through spatially varying retarders**. The output Stokes field of a first-order Néel-type Skyrmion after passing through a retarder with spatially varying axis and retardance. The overall Jones matrix is given by equation (16), with the distribution of *Δ* given by contour plots in the innermost ring and the axis distribution represented by ellipses in the second innermost ring. The output Stokes field is shown in the second outermost ring and the output error as defined in equation (18) is shown in the outermost ring. The Skyrmion numbers are computed using a central difference for partial derivatives and the ‘nquad’ function from SciPy for integration. Note that machine epsilon for double-precision floating-points is of the order 10^−16^

In all simulations, we take *U* to be the open ball of radius *π*/2 centered at the origin, $\psi = \psi_{2} \circ \psi_{1}$ the composition of ${\psi }_{1}(r\cos \theta ,r\sin \theta )=(\tan (r)\cos \theta ,\tan (r)\sin \theta )$ with the inverse stereographic projection from the south pole,

17 $${\psi }_{2}(x,y)=\frac{1}{1+{x}^{2}+{y}^{2}}\left(2x,2y,1-{x}^{2}-{y}^{2}\right)$$

and standard Néel-type Skyrmions (see Supplementary Note 6) as inputs. A central difference is used to compute the partial derivatives of the output Skyrmion field, and an adaptive quadrature rule is used for numerical integration (the ‘nquad’ function from the SciPy library). Figure 4 shows the output Stokes field, retarder axis, and retardance used in simulations. A relative error defined by

18 $$\,\text{error}\,=\left\vert \frac{{\rm{Computed}}\,{\rm{Skyrmion}}\,{\rm{Number}}-{\rm{Initial}}\,{\rm{Skyrmion}}\,{\rm{Number}}}{{\rm{Initial}}\,{\rm{Skyrmion}}\,{\rm{Number}}}\right\vert$$

is also shown. From the figure, we see that numerical error lies in the range 10^−8^ to 10^−12^, which is strong evidence for the validity of our results.

### Spatially varying diattenuators

Consider a spatially varying horizontal linear diattenuator given by the Mueller matrix^54^

19 $$D=\frac{{p}_{1}^{2}}{1+\cos \kappa }\left(\begin{array}{cccc}1&\cos \kappa &0&0\\ \cos \kappa &1&0&0\\ 0&0&\sin \kappa &0\\ 0&0&0&\sin \kappa \end{array}\right)$$

where $\cos \kappa =({p}_{1}^{2}-{p}_{2}^{2})/({p}_{1}^{2}+{p}_{2}^{2})$ is the diattenuation, *p*_1_≥*p*_2_≥0 the principal amplitude coefficients, and spatial dependence suppressed for clarity. Note that, unlike a retarder, the output polarization state through a diattenuator depends on the degree of polarization of the input. If we let $P: U \longrightarrow [0, 1]$ be the spatially varying incident degree of polarization and take $\kappa: U \longrightarrow [0, \pi/2]$, our approach shows that provided *P* and *κ* are smooth, compactifiable in a compatible way with the incident field (duality condition), and satisfy $\cos \tilde{\kappa }(x) < \tilde{P}(x)$ for all *x* ∈ *X*, the Skyrmion number of the field is preserved.

To see why this is true, note first that we may write

20 $${{\mathcal{S}}}^{{\prime} }=f({\mathcal{S}},P,\kappa )=\frac{1}{L({\mathcal{S}},P,\kappa )}\left(\begin{array}{c}\cos \kappa +P{s}_{1}\\ P{s}_{2}\sin \kappa \\ P{s}_{3}\sin \kappa \end{array}\right)$$

where

21 $$L({\mathcal{S}},P,\kappa )=\sqrt{{(1+P{s}_{1}\cos \kappa )}^{2}+({P}^{2}-1){\sin }^{2}\kappa }$$

One important technicality is that for the output polarization state to be well-defined, we require $L({\mathcal{S}},P,\kappa )\ne 0$. Noting that $0\le P,\cos \kappa \le 1$, it is clear that for fixed *P* and *κ*, ${(1+P{s}_{1}\cos \kappa )}^{2}+({P}^{2}-1){\sin }^{2}\kappa$ is minimum when $s_{1} = -1$. In which case, ${L}^{2}={(P-\cos \kappa )}^{2}$. Therefore, $f({\mathcal{S}},P,\kappa )$ is well-defined provided $P\ne \cos \kappa$.

Now, let $\bar{f}:{S}^{2}\longrightarrow {S}^{2}$ be the map

22 $$\bar{f}(s)=f(s,1,\pi /2)=s$$

If $\cos \tilde{\kappa }(x) < \tilde{P}(x)$ for all *x* ∈ *X*, we may construct a continuous homotopy $H: X \times [0, 1] \longrightarrow S^2$ from ${\tilde{{\mathcal{S}}}}^{{\prime} }$ to ${\bar{f}}\,{\circ} \,{\tilde{\mathcal{S}}}$ by

23 $$H(x,t)=f(\tilde{{\mathcal{S}}}(x),(1-t)\tilde{P}(x)+t,(1-t)\tilde{\kappa }(x)+\pi t/2)$$

where this is well-defined because $\cos ((1-t)\tilde{\kappa }(x)+\pi t/2)\le \cos \tilde{\kappa }(x) < \tilde{P}(x)\le (1-t)\tilde{P}(x)+t$ for all (*x*, *t*) ∈ *X* × [0, 1]. By further application of the Whitney Approximation Theorem, we also have that ${\tilde{{\mathcal{S}}}}^{{\prime} }$ and ${\bar{f}}\,{\circ}\, {\tilde{\mathcal{S}}}$ are smoothly homotopic. Then

24 $$\,{\text{deg}}\,{{\mathcal{S}}}^{{\prime} }:=\,{\text{deg}}\,{\tilde{{\mathcal{S}}}}^{{\prime} }=\,{\text{deg}}\,({\bar{f}}\,{\circ}\, {\tilde{\mathcal{S}}})=\,{\text{deg}}\,({\bar{f}})\,{\text{deg}}\,({\tilde{\mathcal{S}}})=\,{\text{deg}}\,({\tilde{\mathcal{S}}})=:\,{\text{deg}}\,{\mathcal{S}}$$

as $\,\text{deg}\,\bar{f}$ is trivially 1.

A heuristic explanation for the required condition comes from the observation that after diattenuation, vertically polarized light has a Stokes vector given by

25 $$\frac{{p}_{1}^{2}}{1+\cos \kappa }\left(\begin{array}{cccc}1&\cos \kappa &0&0\\ \cos \kappa &1&0&0\\ 0&0&\sin \kappa &0\\ 0&0&0&\sin \kappa \end{array}\right)\left(\begin{array}{c}1\\ -P\\ 0\\ 0\end{array}\right)\propto \left(\begin{array}{c}1-P\cos \kappa \\ \cos \kappa -P\\ 0\\ 0\end{array}\right)$$

Observe that for this to remain vertically polarized, we require $\cos \kappa -P < 0$, or equivalently, $\cos \kappa < P$. As a consequence, if $\tilde{P}(x) > \cos \tilde{\kappa }(x)$ for some *x* ∈ *X*, by the smoothness of $\tilde{P}$ and $\tilde{\kappa }$, we can find some open set *V* ⊆ *X* such that $\tilde{P}(x) > \cos \tilde{\kappa }(x)$ for all *x* ∈ *V*. We can then design an input Skyrmion field where vertically polarized light appears only in that region *ψ*^−1^(*V* ∩ *ψ*(*U*)). The output Skyrmion field will then be without vertically polarized light and, as a non-surjective map into the Poincaré sphere, is trivially degree 0.

**Fig. 5:**
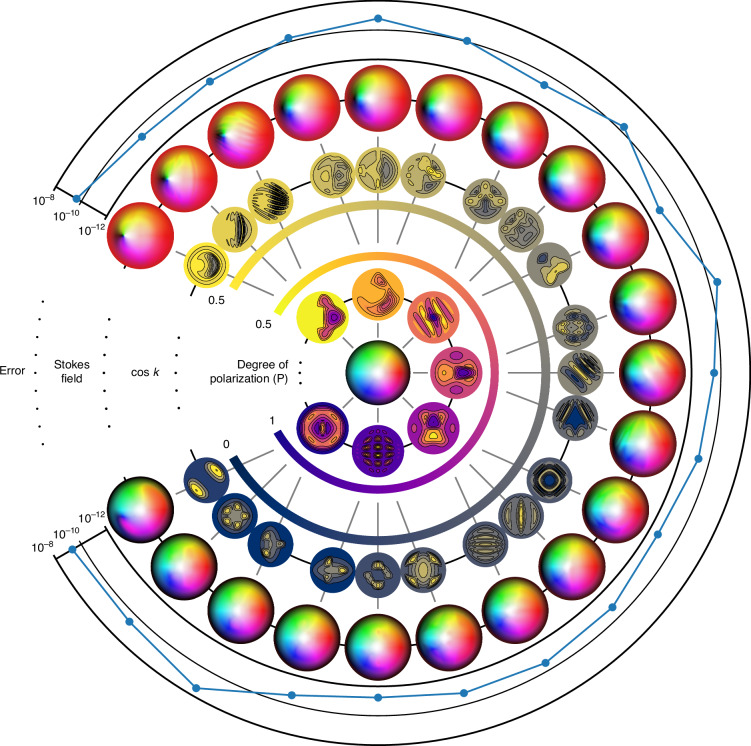
**Skyrmions through spatially varying diattenuators**. The output Stokes field of a first-order Néel-type Skyrmion with spatially varying degree of polarization after passing through a horizontal linear diattenuator with spatially varying diattenuation. This figure follows a similar structure as Fig. 4, where the spatial varying parameters *P* and *κ* are represented by contour plots in the two innermost rings. The Skyrmion numbers are computed using a central difference for partial derivatives and the ‘nquad’ function from SciPy for integration

Figure 5 shows the numerical error in computations for different choices of spatially varying degree of polarization and diattenuation. As in the case of spatially varying retarders, the output Skyrmion number is close to the input value of 1, with errors lying in the range 10^−8^ to 10^−12^.

### Non-depolarizing Systems

In the context of general spatially varying non-depolarizing systems, the singular value decomposition allows us to view the system Jones matrix at every point as a horizontal linear diattenuator sandwiched between two retarders. If, furthermore, this decomposition can be done in a smooth manner, we essentially arrive at a cascade of the two cases presented above and can directly apply the proven conditions. As an application of this, consider the case of a homogeneous non-depolarizing system where such a smooth decomposition is trivially available. Since the constant map extends to a smooth map on *X*, and retarders do not alter the degree of polarization of light, our previous results completely characterize the effects of a homogenous non-depolarizing system on the Skyrmion number of an incoming field in terms of the incident degree of polarization *P* and the diattenuation of the system $\cos \kappa$, namely

26 $$\,{\text{deg}}\,{{\mathcal{S}}}^{{\prime} }=\left\{\begin{array}{rcl}0&&{\rm{if}}\,{\tilde{P}}(x) \,< \,\cos \kappa \,\,{\text{for}}\, {\text{all}}\,\,x\in X\\ \,{\text{deg}}\,{\mathcal{S}}&&{\rm{if}}\,{\tilde{P}}(x) > \cos \kappa \,\,{\text{for}}\, {\text{all}}\,\,x\in X\end{array}\right.$$

In the extreme case of fully polarized light, the above condition can be elegantly restated in terms of the system’s 2^nd^ principal amplitude coefficient,

27 $$\,\text{deg}\,{{\mathcal{S}}}^{{\prime} }=\,\text{deg}\,{\mathcal{S}}\quad \quad {\rm{if}}\,{p}_{2} > 0$$

We, therefore, arrive at the powerful result that for fully polarized light passing through a homogeneous non-depolarizing system, the Skyrmion number remains protected unless the system’s extinction ratio is simultaneously infinite.

In practice, things are not so simple, especially when the system’s extinction ratio is high. More specifically, the effects of a horizontal linear diattenuator on the Poincaré sphere become more and more degenerate as diattenuation increases, where a large proportion of the 2-sphere gets mapped onto a small region about (1, 0, 0) while a small region about (−1, 0, 0) is stretched to cover almost the entire sphere. Qualitatively, this results from the fact that when one eigenstate is attenuated significantly less than the other, its polarization state will dominate the output unless its intensity is also substantially lower. From an experimental perspective, this degeneracy leads to two fundamental difficulties. The first difficulty relates to the limitations of polarimetry at low intensities. Since at high diattenuation, the spatial variation of the output field’s polarization state is concentrated in the region of greatest intensity loss, achieving the necessary signal-to-noise ratio to accurately capture this spatial variation is a significant challenge. Secondly, even with perfect measurements, the finite spatial resolution of cameras will still give rise to unavoidable errors when computing the discrete derivatives and numerical integration needed for the Skyrmion number.

These considerations are, of course, not limited to the fully polarized case. In fact, one can visually observe this degeneracy in the upper left quadrant of Fig. 5, where the vertically polarized states become more and more condensed as the incident degree of polarization approaches diattenuation. For a more detailed discussion of these matters, refer to Supplementary Note 7.

The challenge of experimentally determining the Skyrmion number through spatial sampling highlights the importance of developing alternative ways of identifying the Skyrmion number from polarimetric measurements. From a communications perspective, fast decoding of the Skyrmion number is also essential in maximizing bit-rate, and performing numerical differentiation and integration at the receiver is unlikely to be optimal. One possible property that can be exploited to achieve fast decoding is the fact that the preimage of any regular value under a degree *n* map is at least of size ∣*n*∣. Using this, we could potentially reduce the evaluation of the complex Skyrmion number integral to a simple bright spot counting problem. A complication with this approach, however, is the fact that there are cases in which it fails — consider, for instance, a degree *n* Skyrmion that wraps around the Poincaré sphere *n* + 1 times in one direction and once in the other. Nevertheless, if we transmit an initial Skyrmion that wraps around the Poincaré sphere exactly *n* times, such as a standard Néel or Bloch-type Skyrmion, the field would have to undergo significant deformations before transitioning into one that would result in errors for every polarization state. Therefore, with an appropriate selection of input and channel, it may be feasible to adopt such an intensity-based strategy.

### Depolarizing media

In the context of depolarizing media, the problem of topological protection is complicated by the effects of scattering on the propagation of the output Skyrmion field. Nevertheless, when scattering is small but non-zero, a Mueller matrix treatment of the problem is still largely valid. Borrowing the symmetric matrix decomposition proposed by Ossikovski^55^, we note that every Mueller matrix *M* belongs to one of the following two categories:

1. Type-I, which can be written *M* = *M*_*J*2_*M*_*Δd*_*M*_*J*1_ where *M*_*J*1_ and *M*_*J*2_ are pure Mueller matrices and
  28 $${M}_{\Delta d}=\left(\begin{array}{cccc}{d}_{0}&0&0&0\\ 0&{d}_{1}&0&0\\ 0&0&{d}_{2}&0\\ 0&0&0&\varepsilon {d}_{3}\end{array}\right)$$
  where 0 ≤ *d*_1_, *d*_2_, *d*_3_ ≤ *d*_0_ and $$\varepsilon :=\det M/| \det M|$$.
2. Type-II, which can be written *M* = *M*_*J*2_*M*_*Δnd*_*M*_*J*1_ where *M*_*J*1_ and *M*_*J*2_ are nonsingular pure Mueller matrices and
  29 $${M}_{\Delta nd}=\left(\begin{array}{cccc}2{a}_{0}&-{a}_{0}&0&0\\ {a}_{0}&0&0&0\\ 0&0&{a}_{2}&0\\ 0&0&0&{a}_{2}\end{array}\right)$$
  where 0≤*a*_2_≤*a*_0_.

As in the non-depolarizing case, we consider the situation where the spatially varying Mueller matrix can be smoothly decomposed into the above forms. The pure Mueller matrices can then be handled by our previously proven results, so we need only concentrate on *M*_*Δd*_ and *M*_*Δnd*_.

In the type-I case, we show that topological protection is achieved provided ${d}_{1},{d}_{2},{d}_{3}:U\longrightarrow {{\mathbb{R}}}_{\ge 0}$ are smooth, compactifiable in a compatible way with the incident field (duality condition), satisfy ${\min }_{x\in X}\{{\tilde{d}}_{1}(x),{\tilde{d}}_{2}(x),{\tilde{d}}_{3}(x)\} > 0$ and *ε* is everywhere constant. To prove this, we first define the smooth map $f:{{\mathbb{R}}}^{3}-\{(0,0,0)\}\longrightarrow {S}^{2}$ by

30 $$f({x}_{1},{x}_{2},{x}_{3})=\frac{1}{\sqrt{{x}_{1}^{2}+{x}_{2}^{2}+{x}_{3}^{2}}}\left(\begin{array}{c}{x}_{1}\\ {x}_{2}\\ {x}_{3}\end{array}\right)$$

so that ${{\mathcal{S}}}^{{\prime} }$ can be written smoothly in terms of *d*_1_, *d*_2_, *d*_3_ and ${\mathcal{S}}$ by

31 $${{\mathcal{S}}}^{{\prime} }(p)=f({d}_{1}(p){\mathcal{S}}(p),{d}_{2}(p){\mathcal{S}}(p),\varepsilon {d}_{3}(p){\mathcal{S}}(p))$$

We now define a continuous homotopy $H: X \times [0, 1] \longrightarrow S^2$ as follows

32 $$H(x,t)=f\left(\left((1-t){\tilde{d}}_{1}(x)+t\right){\tilde{\mathcal{S}}}_{1}(x),\left((1-t){\tilde{d}}_{2}(x)+t\right){\tilde{\mathcal{S}}}_{2}(x),\left((1-t){\tilde{d}}_{3}(x)+t\right)\varepsilon {\tilde{\mathcal{S}}}_{3}(x)\right)$$

Notice that since ${\tilde{d}}_{1}(x)$, ${\tilde{d}}_{2}(x)$, ${\tilde{d}}_{3}(x) > 0$ for all *x* ∈ *X*, $(1-t){\tilde{d}}_{i}(x)+t > 0$ for all *x* ∈ *X*, *t* ∈ [0, 1] and *i* = 1, 2, 3. Therefore, *H*(*x*, *t*) is well-defined. Moreover, $H(x,0)={\tilde{{\mathcal{S}}}}^{{\prime} }(x)$ and $H(x,1)=\tilde{{\mathcal{S}}}(x)$. Hence, by the usual Whitney Approximation Theorem argument, ${\tilde{{\mathcal{S}}}}^{{\prime} }$ and $\tilde{{\mathcal{S}}}$ are smoothly homotopic and

33 $$\,{\text{deg}}\,{{\mathcal{S}}}^{{\prime} }:=\,{\text{deg}}\,{\tilde{\mathcal{S}}}^{{\prime} }=\,{\text{deg}}\,{\tilde{\mathcal{S}}}=:\,{\text{deg}}\,{\mathcal{S}}$$

**Fig. 6:**
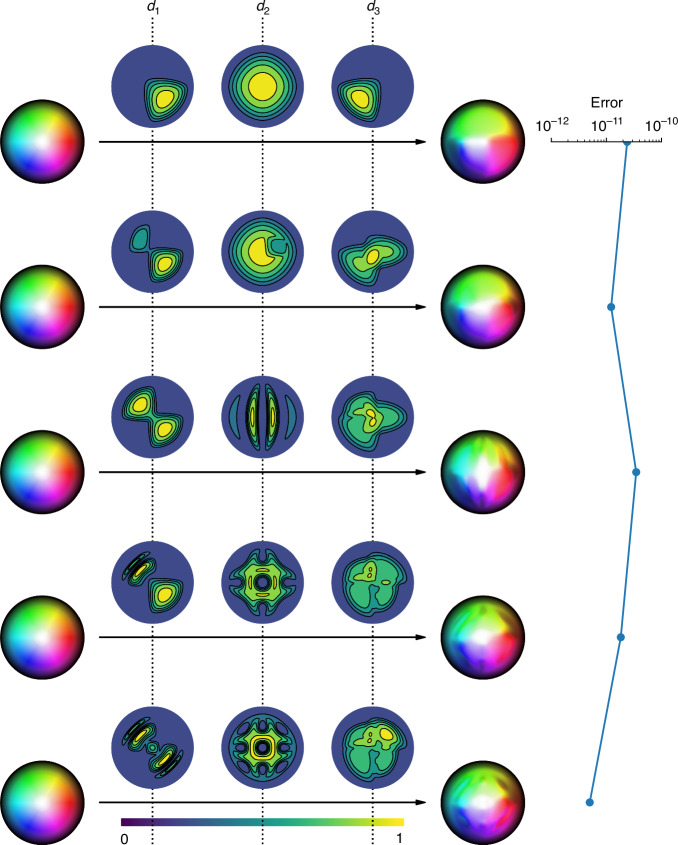
**Skyrmions through spatially varying depolarizers**. The output Stokes field of a first-order Néel-type Skyrmion after passing through a type-I depolarizing media with spatially varying parameters *d*_1_, *d*_2_, *d*_3_ and *ε* = 1. Note from equation (31) that the output Stokes field is independent of the incident degree of polarization. This figure therefore does not include the distribution of *P*. The Skyrmion numbers are computed using a central difference for partial derivatives and the ‘nquad’ function from SciPy for integration

Figure 6 illustrates the effects of different spatially varying type-I depolarizers on a first-order Néel-type Skyrmion and the associated numerical error in simulation.

In the type-II case, the induced transformation of *M*_*Δnd*_ on the Poincaré sphere is given by

34 $$\left(\begin{array}{c}{s}_{1}\\ {s}_{2}\\ {s}_{3}\end{array}\right)\mapsto \frac{1}{\sqrt{{a}_{0}^{2}+{a}_{2}^{2}{P}^{2}({s}_{2}^{2}+{s}_{3}^{2})}}\left(\begin{array}{c}{a}_{0}\\ {a}_{2}P{s}_{2}\\ {a}_{2}P{s}_{3}\end{array}\right)$$

where *P* is the incident degree of polarization. Since 0≤*a*_0_, any Stokes vector with a negative *s*_1_ component is never in the image of this map. Therefore, the output Stokes field of a spatially varying type-II depolarizing media is never surjective, and hence of degree zero.

## Supplementary information

Supplementary Information for Topological Protection of Optical Skyrmions through Complex Media

## Data Availability

The data that supports the plots within this paper and other findings of this study are available from the corresponding author upon reasonable request.
