# Supplementary material for: Topological protection of optical skyrmions through complex media
Source: Light Sci Appl. 2024 Nov 22;13:314. doi: 10.1038/s41377-024-01659-z (PMC11582597; doi:10.1038/s41377-024-01659-z)
Supplement: Supplementary file 1 — Supplementary Information for Topological Protection of Optical Skyrmions through Complex Media [file 41377_2024_1659_MOESM1_ESM.pdf]

Supplementary Information for
Topological Protection of Optical Skyrmions through Complex Media

An Aloysius Wang[1,*], Zimo Zhao[1], Yifei Ma[1], Yuxi Cai[1], Runchen Zhang[1], Xiaoyi Shang[1], Yunqi Zhang[1], Ji Qin[1], Zhi Kai Pong[1], Tádé Marozsák[1], Binguo Chen[2], Honghui He[2], Lin Luo[3], Martin J Booth[1], Steve J Elston[1], Stephen M Morris[1], and Chao He[1,*]

[1]Department of Engineering Science, University of Oxford, Parks Road, Oxford, OX1 3PJ, UK
[2]Guangdong Research Center of Polarization Imaging and Measurement Engineering Technology, Institute of Biopharmaceutical and Health Engineering, Tsinghua Shenzhen International Graduate School, Tsinghua University, Shenzhen 518055, China
[3]College of Engineering, Peking University, Beijing 100871, China
[*]Corresponding authors: an.wang@stcatz.ox.ac.uk, aw6609@princeton.edu; chao.he@eng.ox.ac.uk

1 Defining the Fractional Skyrmion

Let U be an open subset of $\mathbb{R}^2$, X a compact, connected, orientable, smooth 2-dimensional manifold and $\psi\colon U \longrightarrow X$ an orientation preserving diffeomorphism onto its image. Then for any smooth map $\alpha\colon U \longrightarrow Y$, we say α is ψ-extendable if

1. $\alpha \circ \psi^{-1}$ extends via continuity to $\overline{\psi(U)}$ and
2. there exists a smooth $F\colon X - \psi(U) \longrightarrow Y$ such that $F|_{\partial\psi(U)} = \alpha \circ \psi^{-1}|_{\partial\psi(U)}$, where the latter is to be understood as the continuous extension of $\alpha \circ \psi^{-1}$ to $\overline{\psi(U)}$.

We then call

$$\tilde{\alpha}_F = \begin{cases} \alpha \circ \psi^{-1}, & x \in \overline{\psi(U)} \\ F, & x \in X - \psi(U) \end{cases} \tag{1}$$

the (ψ, F)-extension of α to X, where $\tilde{\alpha}_F$ is continuous by a gluing argument and piecewise smooth by construction.

Now suppose $\mathcal{S}\colon U \longrightarrow S^2$ is ψ-extendable and let $\tilde{\mathcal{S}}_F$ be a (ψ, F)-extension. Then $\tilde{\mathcal{S}}_F$, as a continuous and piecewise smooth function between compact, connected, orientable 2-dimensional manifolds, can be assigned a degree

$$\deg \tilde{\mathcal{S}}_F = \int_X \tilde{\mathcal{S}}_F^* \omega_0 = \mathrm{SkyN}(\mathcal{S}) + \int_{X-\psi(U)} F^* \omega_0. \tag{2}$$

Notice that this is a strict generalization of our previous notion of the Skyrmion, where F is uniquely defined by continuity whenever $\psi(U)$ is dense so that $\tilde{\mathcal{S}}$ agrees with its only possible (ψ, F)-extension $\tilde{\mathcal{S}}_F$.

2 Interpreting Fractional Skyrmions

In this section, we discuss two ways in which one can understand the definition of fractional Skyrmions given in this paper. To begin, as an example of the extension procedure presented, consider the situation $U = B_R(0)$. In this case, we may take $\psi(x, y) = \varphi_N^{-1}(x/R, y/R)$ where φ_N is the stereographic projection map from the south pole so that U is mapped to the upper hemisphere $S^+ = \{(x^1, x^2, x^3) \in S^2 \colon x^3 > 0\} \subseteq X = S^2$ as shown in Fig. S1. Now suppose $\mathcal{S} \circ \psi^{-1}$ extends via continuity to $\overline{S^+}$ and let $\partial\tilde{\mathcal{S}}$ be this

extension when restricted to equator ∂S^+. Then $\partial\tilde{\mathcal{S}}\colon \partial S^+ \cong S^1 \longrightarrow S^2$, as a non-surjective map into S^2, is null-homotopic. For example, if $-q \in S^2$ is not in the image of $\partial\tilde{\mathcal{S}}$, then $H\colon \partial S^+ \times [0,1] \longrightarrow S^2$ given by

$$H(\theta,t) = \frac{(1-t)\partial\tilde{\mathcal{S}}(\theta) + tq}{\|(1-t)\partial\tilde{\mathcal{S}}(\theta) + tq\|} \tag{3}$$

is a possible null-homotopy between $\partial\tilde{\mathcal{S}}$ and the constant map taking value q. In general, let $H\colon \partial S^+ \times [0,1] \longrightarrow S^2$ be a homotopy from $\partial\tilde{\mathcal{S}}$ to some $q \in S^2$. We may then use this null-homotopy to extend $\mathcal{S}\circ\psi^{-1}$ to the lower hemisphere $S^- = \{(x_1,x_2,x_3) \in S^2 \colon x_3 \leq 0\}$ by $F\colon S^- \longrightarrow S^2$,

$$F\left(\sqrt{1-x_3^2}\cos\theta, \sqrt{1-x_3^2}\sin\theta, x_3\right) = H(\theta, -x_3), \tag{4}$$

noting that this is well-defined as H descends onto the quotient $\partial S^+ \times [0,1]/\sim$ given by the equivalence relation $(\theta_1, 1) \sim (\theta_2, 1)$ for all $\theta_1, \theta_2 \in \partial S^+$, and this space is clearly homeomorphic to S^- via $[\theta, z] \mapsto (\sqrt{1-z^2}\cos\theta, \sqrt{1-z^2}\sin\theta, -z)$. Conversely, given any well-defined F, equation (4) defines a null-homotopy of $\partial\tilde{\mathcal{S}}$. Therefore, in this context, we may think of a (ψ, F)-extension as a specific choice of null-homotopy of $\partial\tilde{\mathcal{S}}$, and the topological character of such fractional Skyrmions is expressed in the restrictions to the functions F that extend them. Fig. S1 shows an example of F constructed with the homotopy defined by equation (3). Note also that a similar approach can be used to extend $\mathcal{S}$ for any connected U, where appropriate null-homotopies can be used to fill in missing holes on $X = S^2$.

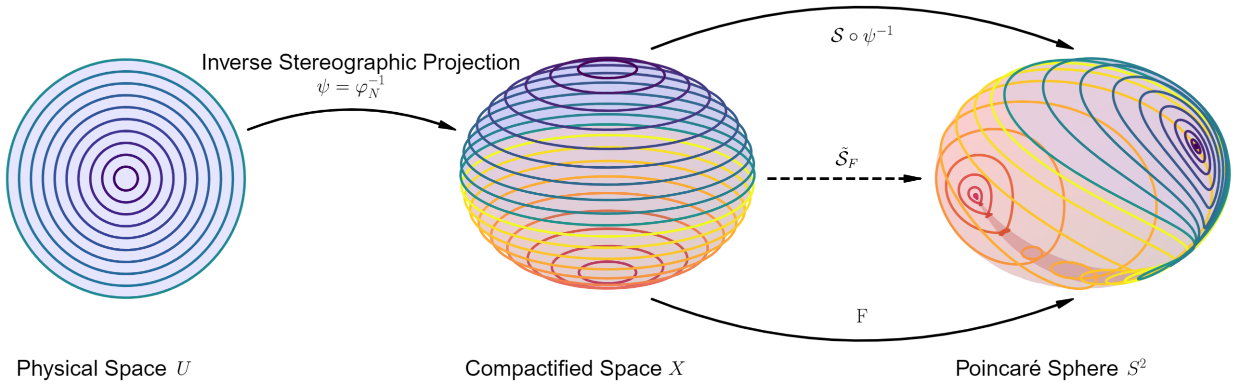

Supplementary Fig. 1: **Geometric interpretation of fractional Skyrmions.** (Left) Physical space U with reference lines. (Middle) Compactified space X with showing reference lines after stereographic projection and fictitious lines corresponding to $X - \psi(U)$. (Right) Poincaré sphere with the image of the reference lines under $\tilde{\mathcal{S}}_F$.

Another way of understanding equation (2) is that the function F exists to provide a correction term $\int_{X-\psi(U)} F^*\omega_0$ which accounts for perturbations of the boundary. This is most evident when considering the propagation of right circularly polarized light through a gradient index lens system as shown in Fig. S2. A most remarkable property of the gradient index lens is that when uniform right circularly polarized light $s = (1,0,0)^T$ couples into a gradient index lens with $\sigma_{\max} = \pi$, the output is a Néel-type Skyrmion of degree 2 (See Supplementary Note 8 for the mathematical details). However, for any $\sigma_{\max} < \pi$, the output field is not compactifiable. Nonetheless, when $\sigma_{\max}$ is close to π, the output field closely resembles that of a Néel-type Skyrmion, and intuitively, its Skyrmion density integrates to a value close to 2. The extension procedure, as shown in Fig. S2, can then be seen as a way of canonically assigning values so that the resultant map from X is simply that of a Néel type Skyrmion. In this way, one can view such "fractional Skyrmions" as a fraction of a regular Skyrmion obtained through extension, hence motivating its name.

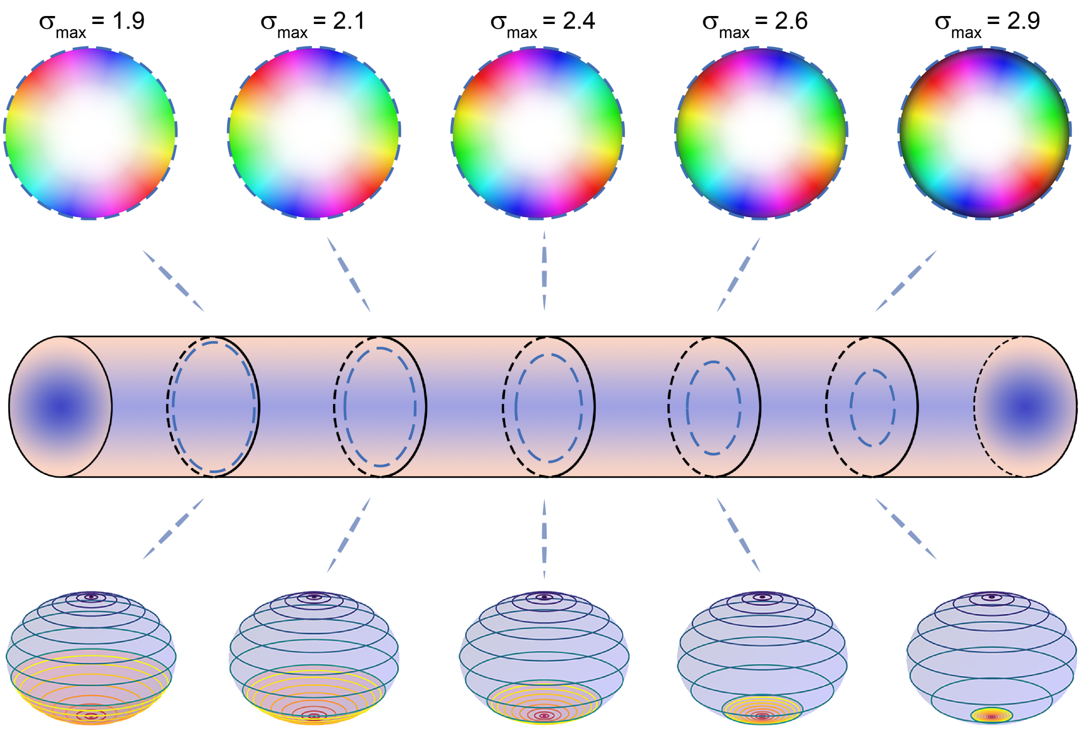

Supplementary Fig. 2: **Fractional Skyrmions in a gradient index lens.** The propagation of right circularly polarized light through a gradient index lens system and the respective extensions of the Stokes field given by equation (3) taking q to be $(0, 0, -1)$. Note we assume that all fields lie within a single pitch of the system.

3 The Topological Protection of Fractional Skyrmions

Through equation (2), we can relate the Skyrmion number of a fractional Skyrmion with the degree of a (ψ, F)-extension of $\mathcal{S}$. In this way, we can make use of the homotopy invariance of the degree to establish robustness results similar to those of regular Skyrmions. We proceed as follows. Let $\mathcal{S}' : U \longrightarrow S^2$ be the output Stokes field and $\tilde{\mathcal{S}}'_{F'}$ a (ψ, F')-extension of $\mathcal{S}'$. If we can construct a homotopy from $\tilde{\mathcal{S}}'_{F'}$ to a map of the form $\bar{f} \circ \tilde{\mathcal{S}}_F$ for some smooth $\bar{f}$ then

$$\begin{aligned}\mathrm{SkyN}(\mathcal{S}') + \int_{X-\psi(U)} F'^*\omega_0 = \deg \tilde{\mathcal{S}}'_{F'} &= \deg(\bar{f} \circ \tilde{\mathcal{S}}_F) \\ &= \deg(\bar{f}) \deg(\tilde{\mathcal{S}}_F) = \deg(\bar{f}) \left(\mathrm{SkyN}(\mathcal{S}) + \int_{X-\psi(U)} F^*\omega_0 \right) \qquad (5)\end{aligned}$$

so that

$$\mathrm{SkyN}(\mathcal{S}') = \deg(\bar{f})\mathrm{SkyN}(\mathcal{S}) + \int_{X-\psi(U)} (\deg(\bar{f})F - F')^*\omega_0. \qquad (6)$$

An important situation where equation (6) simplifies is when $(\mathcal{S}' \circ \psi^{-1})(x) = (f \circ \mathcal{S} \circ \psi^{-1})(x)$ for every $x \in \partial\psi(U)$ where $f(s) = Rs$ for some $R \in SO(3)$. Note here that we understand $\mathcal{S} \circ \psi^{-1}$ and $\mathcal{S}' \circ \psi^{-1}$ in the previous equation as continuous extensions onto $\overline{\psi(U)}$, and whose existence is guaranteed by the conditions in our definition of ψ-extendability. One can then readily verify that $f^*\omega_0 = \omega_0$, so if we take $F' = f \circ F$,

$$\int_{X-\psi(U)} F'^*\omega_0 = \int_{X-\psi(U)} F^*(f^*\omega_0) = \int_{X-\psi(U)} F^*\omega_0, \qquad (7)$$

which implies

$$\mathrm{SkyN}(\mathcal{S}') = \deg(\bar{f})\mathrm{SkyN}(\mathcal{S}) + \big(\deg(\bar{f}) - 1\big)\int_{X-\psi(U)} F^*\omega_0. \tag{8}$$

With the simplification above, we may arrive at similar topological protection results for fractional Skyrmions as stated below.

1. A spatially varying retarder $e^{i\phi(p)}J(p)$ preserves the Skyrmion number whenever J is smooth and $(J \circ \psi^{-1})(x)$ is constant for all $x \in \partial\psi(U)$.

2. The Skyrmion number of a field with spatially varying degree of polarization P impinging on a spatially varying diattenuator with diattenuation $\cos\kappa$ remains unchanged if P, $\cos\kappa$ are smooth and $P \circ \psi^{-1}|_{\partial\psi(U)} = 1$, $\kappa \circ \psi^{-1}|_{\partial\psi(U)} = \pi/2$.

3. The Skyrmion number of a field with spatially varying degree of polarization P impinging on a spatially varying type-I depolarizer remains unchanged if P, d_1, d_2, d_3 are smooth, $\varepsilon = 1$ and $d_1 \circ \psi^{-1}|_{\partial\psi(U)} = d_2 \circ \psi^{-1}|_{\partial\psi(U)} = d_3 \circ \psi^{-1}|_{\partial\psi(U)}$.

Notice that these are somewhat weaker conditions than those established for ordinary Skyrmions, and this is what we expect. By broadening the notion of the Skyrmion, a larger class of functions is considered, and hence, the transformations that leave the Skyrmion number of every field unchanged necessarily become more restrictive.

4 The Integral Formula for Skyrmion Number

To evaluate the pullback

$$\mathcal{S}^*\omega_0 = \frac{1}{4\pi}(\iota \circ S)^*(x^1 dx^2 \wedge dx^3 - x^2 dx^1 \wedge dx^3 + x^3 dx^1 \wedge dx^2), \tag{9}$$

let $\iota \circ \mathcal{S} = S = (S^1, S^2, S^3)$ so that $x^i \circ S = S^i$, $i = 1, 2, 3$. Then, by direction computation,

$$\begin{aligned}(\iota \circ \mathcal{S})^*(x^1 dx^2 \wedge dx^3) &= S^1\left(\frac{\partial S^2}{\partial x}dx + \frac{\partial S^2}{\partial y}dy\right) \wedge \left(\frac{\partial S^3}{\partial x}dx + \frac{\partial S^3}{\partial y}dy\right)\\ &= S^1\left(\frac{\partial S^2}{\partial x}\frac{\partial S^3}{\partial y} - \frac{\partial S^2}{\partial y}\frac{\partial S^3}{\partial x}\right) dx \wedge dy. \end{aligned}\tag{10}$$

Likewise,

$$\begin{aligned}(\iota \circ S)^*(-x^2 dx^1 \wedge dx^3) &= -S^2\left(\frac{\partial S^1}{\partial x}dx + \frac{\partial S^1}{\partial y}dy\right) \wedge \left(\frac{\partial S^3}{\partial x}dx + \frac{\partial S^3}{\partial y}dy\right)\\ &= S^2\left(\frac{\partial S^3}{\partial x}\frac{\partial S^1}{\partial y} - \frac{\partial S^3}{\partial y}\frac{\partial S^1}{\partial x}\right) dx \wedge dy. \end{aligned}\tag{11}$$

and

$$\begin{aligned}(\iota \circ S)^*(x^3 dx^1 \wedge dx^2) &= S^3\left(\frac{\partial S^1}{\partial x}dx + \frac{\partial S^1}{\partial y}dy\right) \wedge \left(\frac{\partial S^2}{\partial x}dx + \frac{\partial S^2}{\partial y}dy\right)\\ &= S^3\left(\frac{\partial S^1}{\partial x}\frac{\partial S^2}{\partial y} - \frac{\partial S^1}{\partial y}\frac{\partial S^2}{\partial x}\right) dx \wedge dy. \end{aligned}\tag{12}$$

Therefore,

$$\begin{aligned}\deg(\mathcal{S}) &= \frac{1}{4\pi}\int_U \left[S^1\left(\frac{\partial S^2}{\partial x}\frac{\partial S^3}{\partial y} - \frac{\partial S^2}{\partial y}\frac{\partial S^3}{\partial x}\right) + S^2\left(\frac{\partial S^3}{\partial x}\frac{\partial S^1}{\partial y} - \frac{\partial S^3}{\partial y}\frac{\partial S^1}{\partial x}\right) + S^3\left(\frac{\partial S^1}{\partial x}\frac{\partial S^2}{\partial y} - \frac{\partial S^1}{\partial y}\frac{\partial S^2}{\partial x}\right)\right] dx \wedge dy\\ &= \frac{1}{4\pi}\iint_U S \cdot \left(\frac{\partial S}{\partial x} \times \frac{\partial S}{\partial y}\right) dxdy. \end{aligned}\tag{13}$$

5 The Whitney Approximation Theorem

The Whitney Approximation Theorem is an important tool that allows us to turn continuous homotopies into smooth homotopies. We give a brief account of the key ideas here. To begin, we make the following definition. Let M and N be smooth manifolds and $A \subseteq M$ a closed subset of M. We say $g\colon M \longrightarrow N$ is smooth on A if there exists an open set $U \supseteq A$ and a smooth function $g_0\colon U \longrightarrow N$ such that $g_0|_A = g|_A$.

The Whitney Approximation Theorem is then the following statement. Let M and N be smooth manifolds, A a closed subset of M, and $g\colon M \longrightarrow N$ a continuous map that is smooth on A. Then there exists a smooth map $f\colon M \longrightarrow N$ homotopic to g such that $f|_A = g|_A$.

An immediate corollary to this is that any smooth maps $f_0, f_1\colon M \longrightarrow N$ that are homotopic are also smoothly homotopic. To see this, let $F\colon M \times [0,1] \longrightarrow N$ be a homotopy connecting f_0 and f_1. We may continuously extend F to a mapping $\tilde{F}\colon M \times \mathbb{R}$ by $\tilde{F}(x,t) = F(x,0)$ when $t \leq 0$ and $\tilde{F}(x,t) = F(x,1)$ when $t \geq 1$. Then $\tilde{F}$ is a continuous map from $M \times \mathbb{R}$ to N. Moreover, $\tilde{F}$ is trivially smooth on $M \times \{0\}$ and $M \times \{1\}$ (We can, for instance, take $\tilde{F}_0\colon M \times (-\epsilon, \epsilon)$, $\tilde{F}_0(x,t) = F(x,0)$, then $\tilde{F}_0$ is clearly smooth and $\tilde{F}_0|_{M\times\{0\}} = \tilde{F}|_{M\times\{0\}}$, and similarly for $M \times \{1\}$). The result then follows by direct application of the Whitney Approximation Theorem.

6 Néel and Bloch-type Skyrmions

Within our framework, we can generalize Néel and Bloch-type Skyrmions to arbitrary domains U in the following way. We begin by constructing a canonical collection of maps $\tilde{\mathcal{S}}$ of arbitrary degree. Note first that if $g\colon S^1 \longrightarrow S^1$ is a map of degree n and $\Sigma\colon \text{Top} \longrightarrow \text{Top}$ the suspension functor, then a simple argument via the Mayer-Vietoris sequence shows that $\Sigma g\colon \Sigma S^1 \cong S^2 \longrightarrow \Sigma S^1 \cong S^2$ is also of degree n. Now, for any $n \in \mathbb{Z}$ and $\gamma \in \mathbb{R}$, let $g_{n,\gamma}\colon S^1 \longrightarrow S^1$ be $g_{n,\gamma}(z) = e^{i\gamma} z^n$. As $\deg g_{n,\gamma}$ is easily checked to be n,

$$\Sigma g_{n,\gamma}(\sin\theta\cos\varphi, \sin\theta\sin\varphi, \cos\theta) = (\sin\theta\cos(n\varphi+\gamma), \sin\theta\sin(n\varphi+\gamma), \cos\theta) \tag{14}$$

is a map from S^2 to S^2 of degree n. We can then use these to define a family of optical fields $S_{n,\gamma}\colon U \longrightarrow S^2$ given by $S_{n,\gamma} = \Sigma g_{n,\gamma} \circ \psi$ such that $\deg S_{n,\gamma} = n$. In this construction, $\gamma = 0$ corresponds to the usual Néel-type Skyrmions while $\gamma = \pi/2$ gives the usual Bloch-type Skyrmions.

7 The Observability of Skyrmion Fields at High Diattenuation

As mentioned in the main text, the degeneracy of the induced mapping on the Poincaré sphere at high diattenuation causes challenges in the numerical evaluation of the Skyrmion number. Graphically, this can be understood from the observation that the Skyrmion density approaches a delta function as diattenuation increases (Fig. S3a), and this imposes additional requirements on spatial resolution akin to the usual Nyquist-like considerations for accurate computation of the Skyrmion number. In a real polarimetry set-up, the finite spatial resolution of cameras means that we only have access to a spatially averaged Stokes vector at specified points, and this limits the ways in which we can compute numerical derivatives and numerical integrals. We can, however, emulate these numerical errors by sampling a theoretical Skyrmion field $N \times N$ times and computing the Skyrmion number integral from these samples. In the following, we use a central difference to estimate the derivative and Simpson's rule for integration. In all simulations, we consider fully polarized incident light passing through a horizontal linear diattenuator.

The results of our simulations are shown in Fig. S3b. As predicted, the grid size needed to obtain a value close to the true Skyrmion number increases with diattenuation. Additionally, for the same grid size, the more complicated third-order Skyrmion fields tend to have poorer accuracy in comparison to their first-order counterparts, reflecting a potential challenge in the detection of high-order Skyrmions. Nonetheless, the results are promising. From a practical perspective, polarisers typically have an extinction ratio in the range $100\colon 1$ to $10^6\colon 1$, which corresponds to $0.002 < \sin\kappa < 0.198$. While the lower end

of this range requires a grid size on the order $N \gtrsim 1000$ for accurate computations, which may well be a challenge to realize in practice, the higher end can be handled by standard-resolution cameras. We stress that these results only account for the accuracy of numerical calculations and not scattering, noise, and other potential experimental imperfections.

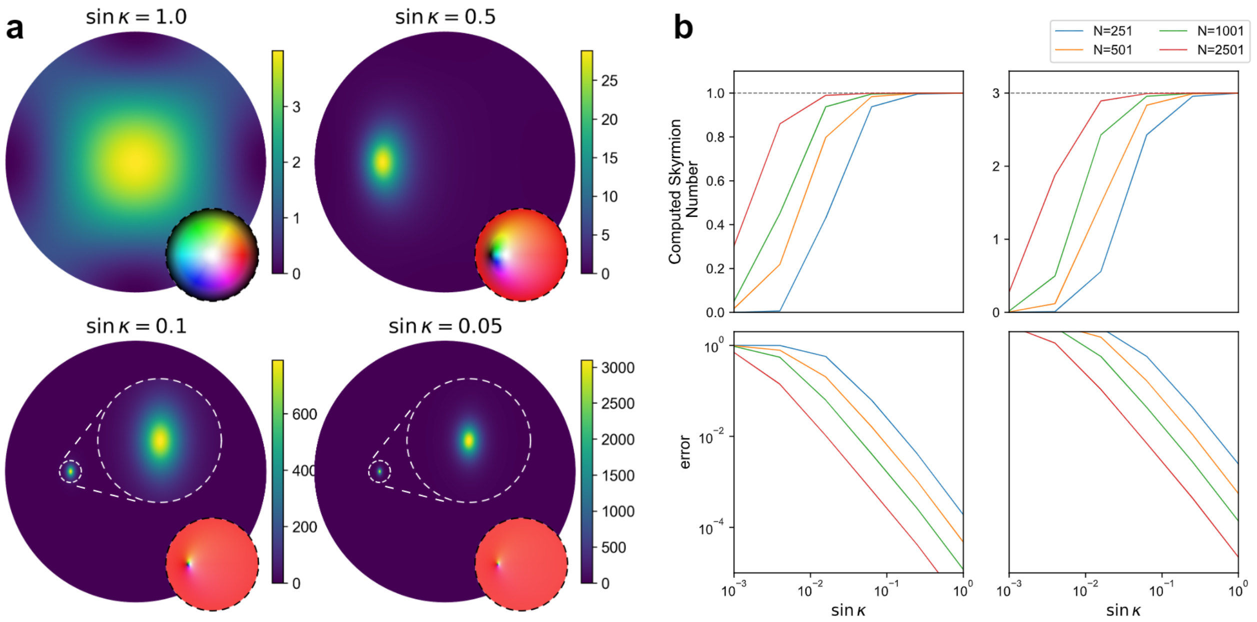

Supplementary Fig. 3: **Simulated Skyrmion number integral at increasing levels of diattenuation. a**, The Skyrmion density and Stokes field of a fully polarized first-order Néel-type Skyrmion after passing through horizontal linear diattenuators of different diattenuation. **b**, Numerically evaluated Skyrmion number and the associated error of a (left) first-order and (right) third-order Néel-type Skyrmion after propagation through horizontal linear diattenuators of different diattenuation.

8 Skyrmion Number Integral in Gradient Index Systems

In this section, we use gradient index lens systems to demonstrate the importance of compactifiability in our definition of the Skyrmion. Consider a gradient index lens, whose effect on polarisation is described by a contraction followed by composition with the smoothly varying but non-compactifiable Mueller matrix

$$M(r,\theta) = \begin{pmatrix} 1 & 0 & 0 & 0 \\ 0 & \cos^2 2\theta + \sin^2 2\theta \cos\sigma(r) & \sin 2\theta \cos 2\theta (1-\cos\sigma(r)) & -\sin 2\theta \sin\sigma(r) \\ 0 & \sin 2\theta \cos 2\theta (1-\cos\sigma(r)) & \sin^2 2\theta + \cos^2 2\theta \cos\sigma(r) & \cos 2\theta \sin\sigma(r) \\ 0 & \sin 2\theta \sin\sigma(r) & -\cos 2\theta \sin\sigma(r) & \cos\sigma(r) \end{pmatrix}, \tag{15}$$

where $\sigma(r)$ smoothly increases from 0 at $r = 0$ to some $\sigma_{\max}$ at the outer circumference $r = r_0$. In the following derivation, we show that for a homogeneous input $s = (s_1, s_2, s_3)$, the Skyrmion number integral evaluates to

$$(1 - \cos\sigma_{\max})s_3, \tag{16}$$

which is not necessarily an integer. Notice further that when $\sigma_{\max} = 2n\pi$, M is constant on the boundary. In this case, the output Skyrmion number evaluates to 0 for all homogeneous inputs, in agreement with the topological protection results established in this paper.

Our main approach to simplifying the Skyrmion number integral is through the use of Stokes' theorem. First, note that with respect to the usual spherical coordinates $(\sin\theta\cos\phi, \sin\theta\sin\phi, \cos\theta)$, we may write

$$\omega_0 = \sin\theta d\theta \wedge d\phi. \tag{17}$$

Letting $N = (0, 0, 1)$ be the north pole and $S = (0, 0, -1)$ be the south pole, notice that on $S^2 - \{N, S\}$, the 1-form $\eta = -\cos\theta d\phi$ is easily seen to satisfy $d\eta = \omega_0$. Therefore, for any $\mathcal{S}\colon U \longrightarrow S^2 - \{N, S\}$,

$$d(\mathcal{S}^*\eta) = S^*(d\eta) = \mathcal{S}^*\omega_0. \tag{18}$$

We can, therefore, define a Skyrmion potential by

$$\mathcal{A} = \mathcal{S}^*\eta = S_3 d\left(\tan^{-1}\left(\frac{S_1}{S_2}\right)\right). \tag{19}$$

Stokes' theorem then gives

$$\mathrm{SkyN}(\mathcal{S}) = \frac{1}{4\pi}\int_U \mathcal{S}^*\omega_0 = \frac{1}{4\pi}\int_U d(\mathcal{S}^*\eta) = \frac{1}{4\pi}\int_{\partial U} \iota^*\mathcal{A}, \tag{20}$$

where $\iota\colon \partial U \hookrightarrow U$ is the inclusion map and ∂U having the induced orientation. Specializing to the gradient index lens, we may take $U = B_{r_0}(0) \subset \mathbb{R}^2 \cong \mathbb{C}$ with its standard orientation. Then for any $\mathcal{S}\colon U \longrightarrow S^2 - \{N, S\}$,

$$\mathrm{SkyN}(\mathcal{S}) = \frac{1}{4\pi}\int_0^{2\pi} S_3(r_0 e^{i\theta})\frac{d}{d\theta}\tan^{-1}\left(\frac{S_1(r_0 e^{i\theta})}{S_2(r_0 e^{i\theta})}\right) d\theta. \tag{21}$$

In the case where S_3 takes values ± 1 at some point $p \in U$. We may still define $\mathcal{A}$ everywhere else and evaluate the Skyrmion number by running a contour around p as in Fig. S4a. The contribution of the singularity at p can then be computed by

$$\begin{aligned} &\lim_{\varepsilon\searrow 0} -\frac{1}{4\pi}\int_0^{2\pi} S_3(p+\varepsilon e^{i\theta})\frac{d}{d\theta}\tan^{-1}\left(\frac{S_1(p+\varepsilon e^{i\theta})}{S_2(p+\varepsilon e^{i\theta})}\right) d\theta \\ &= -\frac{S_3(p)}{2} \times \lim_{\varepsilon\searrow 0}\left(\text{winding number of } (S_2(p+\varepsilon e^{i\theta}), S_1(p+\varepsilon e^{i\theta})) \text{ about } (0,0)\right). \end{aligned} \tag{22}$$

With this machinery on hand, we can break the problem down into three separate cases. We focus on the situation where $0 < \sigma_{\max} \le \pi$, noting that for $\sigma_{\max} > \pi$, U can be divided down into the disk given by $0 \le r \le \sigma^{-1}(\pi)$ and annuli given by $\sigma^{-1}(\pi) \le r \le \sigma^{-1}(2\pi)$, …, $\sigma^{-1}(k\pi) \le r \le r_0$, which can be individually handled in the manner described below.

8.1 Case I: $s_3 = \pm 1$

When $s_3 = \pm 1$, the output Stokes field is given by

$$\iota \circ \mathcal{S} = \begin{pmatrix} S_1 \\ S_2 \\ S_3 \end{pmatrix} = \begin{pmatrix} -s_3 \sin\sigma \sin 2\theta \\ s_3 \sin\sigma\cos 2\theta \\ s_3\cos\sigma \end{pmatrix} \tag{23}$$

so that $\mathcal{A} = -2s_3\cos\sigma d\theta$, and where this is well-defined for all $0 < r \le r_0$ when $\sigma_{\max} < \pi$ and $0 < r < r_0$ when $\sigma_{\max} = \pi$. We may therefore directly apply equation (20) to get

$$\begin{aligned} \mathrm{SkyN}(\mathcal{S}) &= \lim_{\varepsilon\searrow 0}\frac{1}{4\pi}\int_0^{2\pi} -2s_3\left(\cos(\sigma(r_0-\varepsilon)) - \cos(\sigma(\varepsilon))\right) d\theta \\ &= -s_3\left(\cos(\sigma(r_0)) - \cos(\sigma(0))\right) = (1-\cos\sigma_{\max})s_3 \end{aligned} \tag{24}$$

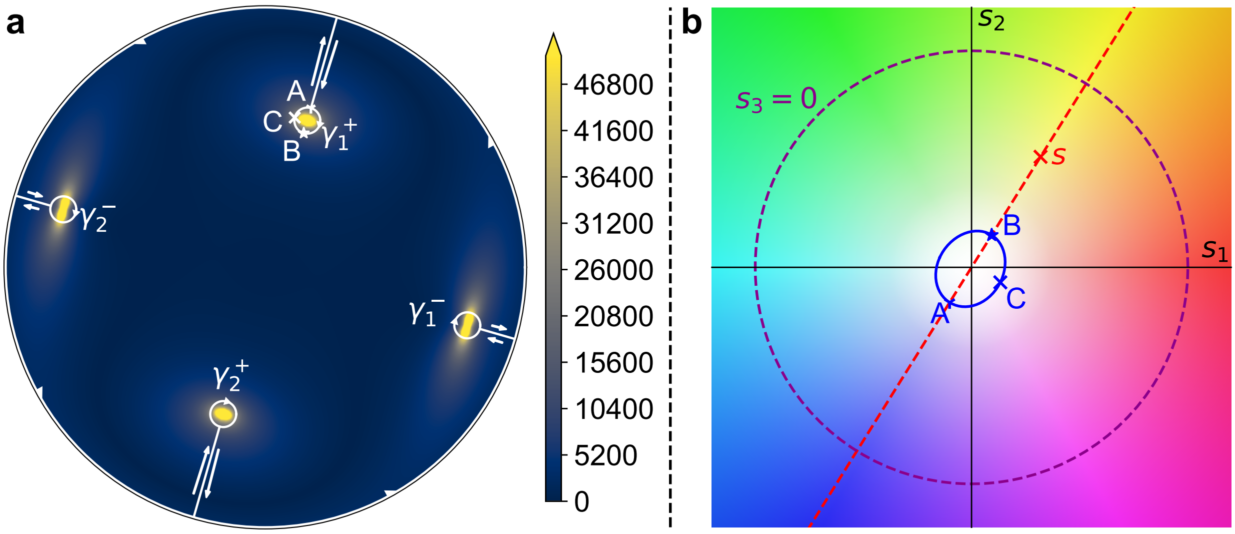

Supplementary Fig. 4: **Contour for Skyrmion number integral.** **a**, Magnitude of $\mathcal{A}(r, \theta)$ for a gradient index lens system with input $(s_1, s_2, s_3) \propto (1, 1, 1)$, $\sigma_{\max} = \pi$ and birefringence quadratic with radius. White lines show a positively oriented contour that can be used to evaluate the Skyrmion number. **b**, The image of γ_1^+ under $\mathcal{S}$ after stereographic projection from the south pole along with distinguished points used to determine the winding number of κ_1^+.

8.2 Case II: $-1 < s_3 < 1$, $\sigma_{\max} = \pi$

Notice the output stokes field when restricted to $r = r_0$ is

$$\begin{pmatrix} S_1(r_0 e^{i\theta}) \\ S_2(r_0 e^{i\theta}) \\ S_3(r_0 e^{i\theta}) \end{pmatrix} = \begin{pmatrix} s_1 \cos 4\theta + s_2 \sin 4\theta \\ s_1 \sin 4\theta - s_2 \cos 4\theta \\ -s_3 \end{pmatrix} \tag{25}$$

Therefore,

$$\frac{1}{4\pi} \int_{\partial U} \iota^* \mathcal{A} = \frac{-s_3}{2} \times (\text{winding number of } (s_1 \sin 4\theta - s_2 \cos 4\theta, s_1 \cos 4\theta + s_2 \sin 4\theta) \text{ about } (0,0)) \tag{26}$$

Setting $\tan \phi = s_1/s_2$, we see that

$$s_1 \sin 4\theta - s_2 \cos 4\theta = -\sqrt{s_1^2 + s_2^2} \cos(4\theta + \phi) \tag{27}$$

$$s_1 \cos 4\theta + s_2 \sin 4\theta = \sqrt{s_1^2 + s_2^2} \sin(4\theta + \phi) \tag{28}$$

so the winding number of $(s_1 \sin 4\theta - s_2 \cos 4\theta, s_1 \cos 4\theta + s_2 \sin 4\theta)$ about $(0, 0)$ is easily seen to be -4, independent of s. Hence

$$\frac{1}{4\pi} \int_{\partial U} \iota^* \mathcal{A} = 2s_3. \tag{29}$$

We now need to account for the points $S_3 = \pm 1$. We first consider $S_3 = 1$. To begin, notice that $M(r, \theta)$ induces a rotation of angle $\sigma(r)$ about the unit axis $(-\cos 2\theta, -\sin 2\theta, 0)$. On the other hand, the required transformation from (s_1, s_2, s_3) to $(0, 0, 1)$ is a rotation about the axis $(s_2, -s_1, 0)$ by an angle $\cos \phi = s_3$. By matching these conditions, we may solve for $S_3 = \pm 1$. However, the advantage of adopting the Skyrmion potential is that we deal with winding numbers, so we need not solve these equations explicitly. Indeed, doing so is impossible without an explicit formula for $\sigma(r)$. The strength of dealing with topological concepts is that a qualitative picture is sufficient.

To see how we may proceed, note first that as $\sigma(r)$ is strictly increasing from 0 to π, $\cos\sigma(r)$ is strictly decreasing from 1 to -1, and there is therefore a single ρ_+ such that $\cos(\sigma(\rho_+)) = s_3$. As for the equation in θ, it is easy to see that there are precisely two solutions, which we denote ξ and $\xi + \pi$. Therefore, we need to determine the winding numbers of the two curves κ_1^+ and κ_2^+ defined by

$$\kappa_1^+(\chi) = (S_2(\gamma_1^+(\chi)), S_1(\gamma_1^+(\chi))), \qquad \gamma_1^+(\chi) = \rho_+ e^{i\xi} + \varepsilon e^{i\chi} \tag{30}$$

$$\kappa_2^+(\chi) = (S_2(\gamma_2^+(\chi)), S_1(\gamma_2^+(\chi))), \qquad \gamma_2^+(\chi) = -\rho_+ e^{-\xi} + \varepsilon e^{i\chi}. \tag{31}$$

We now proceed with a geometric argument. First, consider the intersection of $(S_1(\gamma_1^+(\xi)), S_2(\gamma_1^+(\xi))$ with the plane spanned by (s_1, s_2, s_3) and $(0,0,1)$ as indicated by the red line in Fig. S4b. For a point on $(S_1(\gamma_1^+(\chi)), S_2(\gamma_1^+(\chi))$ to land on this plane, we necessarily require a rotation about the axis $(s_2, -s_1, 0)$, and this corresponds to having $\arg(\gamma_1^+(\chi)) = \xi$ or $\xi + \pi$, or correspondingly, $\chi = \xi$ and $\chi = \xi + \pi$.

As we are concerned with the limit $\varepsilon \searrow 0$, there is no loss of generality in assuming ε is smaller than ρ_+. From this, we have the following. When $\chi = \xi$, $\gamma_1^+(\xi) = (\rho_+ + \varepsilon)e^{i\xi}$, so the retardance at that point satisfies $\sigma(\rho_+ + \varepsilon) > \sigma(\rho_+)$. Therefore, $(S_1(\gamma_1^+(\xi)), S_2(\gamma_1^+(\xi)))$ ends up in the direction of $-(s_1, s_2)$ from the origin. This is illustrated by the point A in Fig. S4b. Conversely, when $\chi = \xi + \pi$, $\gamma_1^+(\xi + \pi) = (\rho_+ - \varepsilon)e^{i\xi}$, so the retardance at that point satisfies $\sigma(\rho_+ - \varepsilon) < \sigma(\rho_+)$, and $(S_1(\gamma_1^+(\xi+\pi)), S_2(\gamma_1^+(\xi+\pi)))$ ends up in the direction (s_1, s_2) from the origin. This is illustrated in Fig. S4b by the point B.

From this, we immediately see that the winding number of κ_1^+ can only be ± 1. To determine the sign, consider a third point $\chi = \xi + \phi$ where $0 < \phi < \pi$ and $|\gamma_1^+(\xi + \phi)| = \rho_+$. Notice that $\arg(\gamma_1^+(\xi + \phi)) > \arg(\gamma_1^+(\xi))$. Therefore, the angle made by the axis of rotation in the $s_1 - s_2$ plane is more positive when $\chi = \xi + \phi$ as compared to $\chi = \xi$. Therefore, $(S_1(\gamma_1^+(\xi + \phi)), S_2(\gamma_1^+(\xi + \phi)))$ lies in the half-plane given by the dividing line $(s_1 t, s_2 t)$ not containing $(s_2, -s_1)$. This is illustrated by the point C in Fig. S4b. From these three distinguished points, it is immediately obvious that the curve $(S_1(\gamma_1^+(\chi)), S_2(\gamma_1^+(\chi)))$ has a winding number of 1, and so κ_1^+ has a winding number of -1. A similar argument shows that κ_2^+ also has a winding number of -1.

The $S_3 = -1$ case follows in an analogous manner, where we can likewise show that the winding number of each respective curve is -1. As a result, the total contribution of the $S_3 = 1$ and $S_3 = -1$ singularities cancel each other out, and overall, we have a Skyrmion number of $2s_3 = (1 + \cos\sigma_{\max})s_3$.

8.3 Case III: $-1 < s_3 < 1$, $0 < \sigma_{\max} < \pi$

Notice first that whenever $0 < \sigma_{\max} < \pi$, the polarization state s is observed only at $r = 0$ while the polarization state $-s$ cannot be observed in the output as the angle of rotation that the gradient index lens induces on the Poincaré is everywhere $< \pi$. This suggests that a change of variables may simplify the problem by reducing the number of singularities that need to be checked. To that end, we define $P = R_c(s)\iota \circ \mathcal{S}$ where R_c is the rotation matrix defined by

$$R_c(s) = \begin{pmatrix} 1 - \frac{s_1^2}{1+s_3} & -\frac{s_1 s_2}{1+s_3} & s_1 \\ \frac{-s_1 s_2}{1+s_3} & 1 - \frac{s_2^2}{1+s_3} & s_2 \\ -s_1 & -s_2 & s_3 \end{pmatrix} \quad \text{so that} \quad R_c(s)^T s = \begin{pmatrix} 0 \\ 0 \\ 1 \end{pmatrix}. \tag{32}$$

If we now consider the potential

$$\mathcal{A}_P = P_3 d\left(\tan^{-1}\left(\frac{P_1}{P_2}\right)\right), \tag{33}$$

then the singular points of $\mathcal{A}_P$ correspond to $P_3 = (0,0,\pm 1)$ or equivalently $S = \pm s$, and by our remark above, $\mathcal{A}_P$ has just one singularity at $r = 0$. Now, to evaluate the integral about ∂U, note first that we

may write

$$P_3\frac{\partial}{\partial\theta}\tan^{-1}\left(\frac{P_1}{P_2}\right)=\frac{P_3}{P_1^2+P_2^2}\left(P_2\frac{\partial P_1}{\partial\theta}-P_1\frac{\partial P_2}{\partial\theta}\right)=\frac{P_3}{1-P_3^2}\left(P_2\frac{\partial P_1}{\partial\theta}-P_1\frac{\partial P_2}{\partial\theta}\right)$$
$$=\left(\frac{1}{1-P_3^2}-\frac{1}{1+P_3}\right)\left(P_2\frac{\partial P_1}{\partial\theta}-P_1\frac{\partial P_2}{\partial\theta}\right) \tag{34}$$

so that

$$\frac{1}{4\pi}\int_0^{2\pi}P_3(r_0e^{i\theta})\frac{d}{d\theta}\tan^{-1}\left(\frac{P_1(r_0e^{i\theta})}{P_2(r_0e^{i\theta})}\right)d\theta$$
$$=\frac{1}{2}\times\Big(\text{winding number of }(P_2(r_0e^{i\theta}),P_1(r_0e^{i\theta}))\text{ about }(0,0)\Big)$$
$$-\frac{1}{4\pi}\int_0^{2\pi}\left(\frac{1}{1+P_3(r_0e^{i\theta})}\right)\left(P_2(r_0e^{i\theta})\frac{dP_1}{d\theta}(r_0e^{i\theta})-P_1(r_0e^{i\theta})\frac{dP_2}{d\theta}(r_0e^{i\theta})\right)d\theta. \tag{35}$$

By direct computation, one may show that

$$\left(\frac{1}{1+P_3(r_0e^{i\theta})}\right)\left(P_2(r_0e^{i\theta})\frac{dP_1}{d\theta}(r_0e^{i\theta})-P_1(r_0e^{i\theta})\frac{dP_2}{d\theta}(r_0e^{i\theta})\right)=-2(1-\cos\sigma_{\max})s_3$$
$$-2\sin\sigma_{\max}(1-\cos\sigma_{\max})\frac{(s_1\sin2\theta-s_2\cos2\theta)^3+(1-s_1^2-s_2^2)(s_1\sin2\theta-s_2\cos2\theta)}{1+\cos\sigma_{\max}+(1-\cos\sigma_{\max})(s_1\cos2\theta+s_2\sin2\theta)^2} \tag{36}$$

Note further that

$$s_1\cos2\theta+s_2\sin2\theta=\sqrt{s_1^2+s_2^2}\cos(2\theta-\phi) \tag{37}$$
$$s_1\sin2\theta-s_2\cos2\theta=\sqrt{s_1^2+s_2^2}\sin(2\theta-\phi) \tag{38}$$

where $\tan\phi=s_2/s_1$. Now, setting $R_s=\sqrt{s_1^2+s_2^2}$, we have,

$$-\frac{1}{4\pi}\int_0^{2\pi}\left(\frac{1}{1+P_3(r_0e^{i\theta})}\right)\left(P_2(r_0e^{i\theta})\frac{dP_1}{d\theta}(r_0e^{i\theta})-P_1(r_0e^{i\theta})\frac{dP_2}{d\theta}(r_0e^{i\theta})\right)d\theta$$
$$=(1-\cos\sigma_{\max})s_3+\frac{\sin\sigma_{\max}(1-\cos\sigma_{\max})}{2\pi}\int_0^{2\pi}\frac{R_s^3\sin^3(2\theta-\phi)+(1-s_1^2-s_2^2)R_s\sin(2\theta-\phi)}{1+\cos\sigma_{\max}+(1-\cos\sigma_{\max})R_s^2\cos^2(2\theta-\phi)}d\theta$$
$$=(1-\cos\sigma_{\max})s_3+\frac{\sin\sigma_{\max}(1-\cos\sigma_{\max})}{2\pi}\int_{-\pi}^{\pi}\frac{R_s^3\sin^3(2\theta)+(1-s_1^2-s_2^2)R_s\sin(2\theta)}{1+\cos\sigma_{\max}+(1-\cos\sigma_{\max})R_s^2\cos^2(2\theta)}d\theta$$
$$=(1-\cos\sigma_{\max})s_3, \tag{39}$$

where the integral in the second last line vanishes by the oddness of the integrand.

Now to account for the singularity at $r=0$, note that (P_2,P_1) is never zero when $r>0$, and therefore, for each $0<r\le r_0$, $H\colon S^1\times[r,r_0]\longrightarrow\mathbb{R}^2-\{(0,0)\}$ given by

$$H(e^{i\theta},\rho)=(P_2(\rho e^{i\theta}),P_1(\rho e^{i\theta})) \tag{40}$$

is a well-defined homotopy from $(P_2(re^{i\theta}),P_1(re^{i\theta}))$ to $(P_2(r_0e^{i\theta}),P_1(r_0e^{i\theta}))$. Consequently, the winding number of $(P_2(re^{i\theta}),P_1(re^{i\theta}))$ about $(0,0)$ is equal to that of $(P_2(r_0e^{i\theta}),P_1(r_0e^{i\theta}))$ about $(0,0)$ for each $r>0$. From this, we have

$$\text{Skyrmion Number}=(1-\cos\sigma_{\max})s_3$$
$$-\frac{1}{2}\times\lim_{\varepsilon\searrow0}\Big(\text{winding number of }(P_2(\varepsilon e^{i\theta}),P_1(\varepsilon e^{i\theta}))\text{ about }(0,0)\Big)$$
$$+\frac{1}{2}\times\Big(\text{winding number of }(P_2(r_0e^{i\theta}),P_1(r_0e^{i\theta}))\text{ about }(0,0)\Big)$$
$$=(1-\cos\sigma_{\max})s_3 \tag{41}$$

From this, we see that the formula

$$\text{SkyN}(\mathcal{S}) = (1 - \cos\sigma_{\text{max}})s_3 \tag{42}$$

holds in all cases.

9 Experiment Assembly

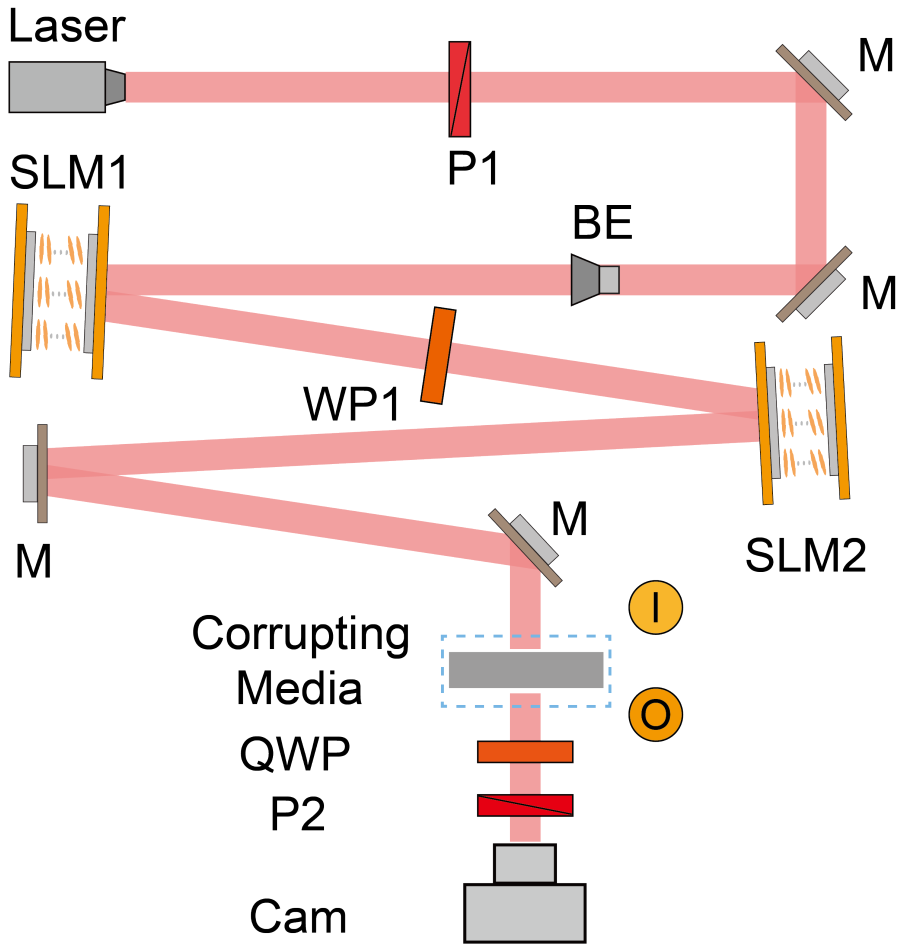

Supplementary Fig. 5: **Experiment assembly.** The complex beam generator and polarimetry configuration adopted in experiments. Components include a He-Ne laser (Melles Griot, 05-LHP-171, 632.8 nm); P1, P2: fixed polarizers (Thorlabs, GL10-A); SLM1, SLM2: spatial light modulators (Hamamatsu, X10468-01); BE: beam expander; WP: fixed waveplate cascade (350-850nm) that achieves a 45° relative rotation between SLMs; QWP: rotating quarter waveplate (Thorlabs, WPQ10M-633) used in polarimetry; Cam: camera (Thorlabs, DCC3240N).

10 Mueller Matrices of Select Experiment Configurations

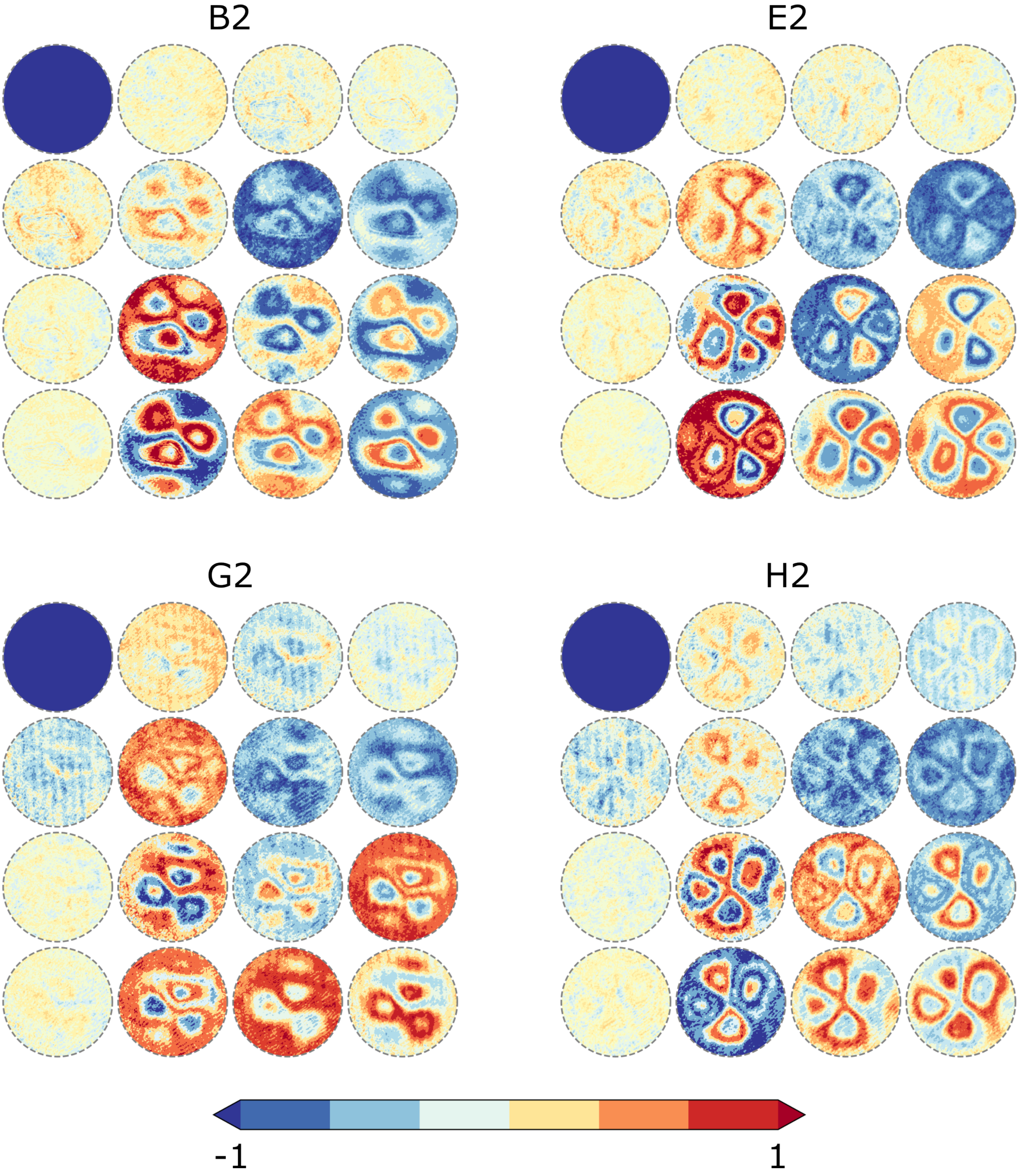

Supplementary Fig. 6: **Mueller matrices of select experiment configurations.** The Mueller matrices of experiment configurations B2, E2, G2, and H2. This figure gives a quantitative characterization of the aberrations and highlights their spatially varying nature.
